# Predictors of cocaine use disorder treatment outcomes: a systematic review

**DOI:** 10.1186/s13643-024-02550-z

**Published:** 2024-05-08

**Authors:** Alba Palazón-Llecha, Beatriz Caparrós, Joan Trujols, Santiago Duran-Sindreu, Francesca Batlle, Mercè Madre, Núria Mallorquí-Bagué

**Affiliations:** 1 Department of Psychiatry, Addictive Behaviours Unit, Hospital de La Santa Creu I Sant Pau, Sant Antoni Maria Claret 167, Pavelló 20, Planta 2, 08025 Barcelona, Spain; 2 Mental Health Research Group, Institut de Recerca Sant Pau (IR Sant Pau), Sant Quintí 77-79, 08041, Barcelona, Spain; 3 Department of Psychology, University of Girona, Edifici Seminari, Campus Barri Vell, Sant Domènech 9, 17004 Girona, Spain; 4 Centro de Investigación Biomédica en Red de Salud Mental (CIBERSAM), Instituto de Salud Carlos III (ISCIII), Monforte de Lemos 3-5, Pavellón 11, Planta 0, 28029 Madrid, Spain

**Keywords:** Cocaine use disorder, Predictors, Treatment outcome, Systematic, Review

## Abstract

**Background:**

Psychosocial approaches are the first-line treatments for cocaine dependence, although they still present high dropout and relapse rates. Thus, there is a pressing need to understand which variables influence treatment outcomes to improve current treatments and prevent dropout and relapse rates. The aim of this study is to explore predictors of treatment retention and abstinence in CUD.

**Methods:**

This systematic review was performed according to the Preferred Reporting Items for Systematic Reviews and Meta-Analyses (PRISMA). We searched three databases—PubMed, PsychINFO and Web of Science—for randomized clinical trials (RCTs) published in English and Spanish from database inception through April 1, 2023. We selected all studies that met the inclusion criteria (adults aged ≥ 18, outpatient treatment, CUD as main addiction, and no severe mental illness) to obtain data for the narrative synthesis addressing cocaine abstinence and treatment retention as main outcome variables. After data extraction was completed, risk of bias was assessed using the Cochrane risk-of-bias tool for randomized trials (RoB-2).

**Results:**

A total of 566 studies were screened, and, of those, 32 RCTs were included in the synthesis. Younger age, more years of cocaine use, and craving levels were significant predictors of relapse and treatment dropout. Fewer withdrawal symptoms, greater baseline abstinence, greater treatment engagement, and more self-efficacy were all predictors of longer duration of abstinence. The role of impulsivity as a predictor of CUD is unclear due to conflicting data, although the evidence generally suggests that higher impulsivity scores can predict more severe addiction and withdrawal symptoms, and earlier discontinuation of treatment.

**Conclusion:**

Current evidence indicates which variables have a direct influence on treatment outcomes, including well-studied cocaine use-related variables. However, additional variables, such as genetic markers, appear to have a high impact on treatment outcomes and need further study.

**Systematic review registration:**

This systematic review is registered at PROSPERO (ID: CRD42021271847). This study was funded by the Spanish Ministry of Science, Innovation and Universities, Instituto Carlos III (ISCIII) (FIS PI20/00929) and FEDER funds and Fundació Privada Hospital de la Santa Creu i Sant Pau (Pla d’acció social 2020).

**Supplementary Information:**

The online version contains supplementary material available at 10.1186/s13643-024-02550-z.

## Introduction

Cocaine use disorder (CUD) is a chronic condition characterized by frequent relapses. This disorder imposes a significant burden on patients, their families and the community. For this reason, treatment services generally need to work with patients over their entire lifetime to prevent drug-related death and/or relapse during personally challenging times. According to data from the European Union, the time interval between the mean age of first cocaine use and first treatment is > 10 years, with 47% of cocaine users in the clinical sample starting treatment for the first time after this period [1]. This finding implies that most cocaine users initiate treatment only after the addiction has become well-established and thus highly resistant to treatment.

According to available evidence, psychosocial approaches are defined as the first-line treatments for CUD. Unlike other illicit substances such as opioids, there is no specific pharmacological treatment for cocaine, which emphasizes the use of psychosocial treatments in addressing this condition [2, 3]. However, psychosocial approaches still present high dropout and relapse rates, thus, there is a pressing need to understand which variables influence treatment outcomes. For this reason, it is important to continue improving psychosocial interventions to reduce the chronicity of the disorder. Contingency management (CM) and cognitive-behavioral therapy (CBT) are the most appropriate approaches for CUD [2, 4–6]. Moreover, there is some evidence to suggest that adding CM to CBT in the treatment of cocaine-related disorders, especially at the beginning of treatment, can help to improve and maintain abstinence at 6-months [3].

Based on the currently available evidence [7], the best predictors of treatment outcomes are 1) treatment retention (measured by urinalysis), 2) craving (measured through the Cocaine Selective Severity Assessment [CSSA]), and 3) impulsivity, regardless of how it is measured [8]. In other words, lower treatment retention rates and higher craving and impulsivity levels predict worse outcomes.

Despite predictive factors of dropout and relapse are relevant to identify deficiencies in cocaine dependence treatment, the last review about this topic was published in 2007 [7]. Therefore, there is a need for a comprehensive update. Our work focuses on exploring all the evidence from published RCTs assessing a wide range of predictors of CUD treatment outcomes from inception until now. This approach has advantages regarding the inclusion of new predictors not previously considered, such as genetic markers to explore new, potentially innovative, ways of personalizing CUD treatment.

The present task involves exploring factors that accumulate substantial evidence that should be incorporated into treatment protocols, as well as those lacking sufficient evidence which warrant exploration to determine their potential relevance in the evolution and prognosis of CUD.

In this context, the aim of the present systematic review was to explore predictors of treatment outcomes in CUD. To perform the review, we searched the main databases to identify all RCTs that have specifically measured predictors of treatment outcomes in CUD.

## Method

### Search strategy

**Fig. 1:**
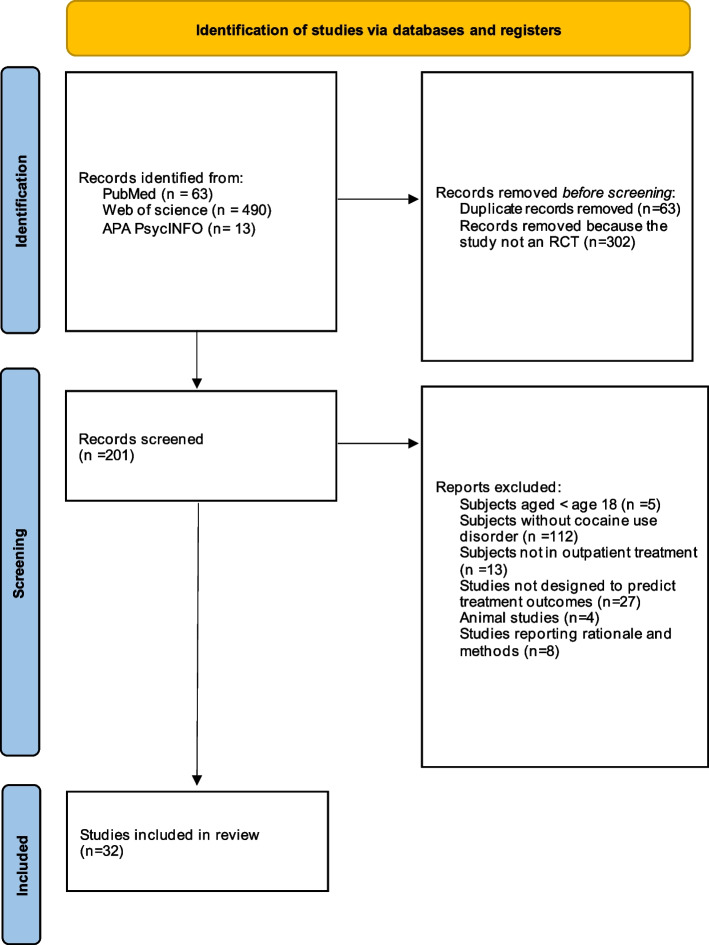
PRISMA flow-chart depicted. 566 records were found and 32 were included in the systematic review

This systematic review was conducted in accordance with the Preferred Reporting Items for Systematic Reviews and Meta-Analyses (PRISMA) reporting guidelines (Fig. 1 and additional files 1 and 2) [9]. This review was registered and is available for consultation at PROSPERO, the international prospective register of systematic reviews of the National Institute for Health Research (registration number: CRD42021271847) on October 14, 2021. We searched three databases—PubMed, APA PsychINFO, and Web of Science—from database inception through April 1, 2023. We searched the PubMed database for clinical trials and RCTs, the APA PsychINFO database for journal articles and clinical trials and the Web of Science for journal articles in the main library. Only articles published in English or Spanish were included. The search strategy was the same for the three databases using terms related to the outcome and the population, as follows: (cocaine)AND(treatment outcome)AND(predictors). The search yielded 63 records from PubMed, thirteen from APA PsychINFO, and 490 from Web of Science (see additional file 3).

### Selection criteria

The inclusion criteria for the studies were as follows: 1) adults ≥ age 18; 2) outpatient treatments, and 3) CUD as the main addiction according to Diagnostic and Statistical Manual of Mental Disorders, Fifth Edition (DSM-5) criteria or to the Mini International Neuropsychiatric Interview (MINI). Exclusion criteria for the studies were: 1) inpatient treatment or treatments other than outpatient treatment and 2) severe mental illness or any main addiction other than CUD. Given the different modalities of treatment settings available (i.e.: inpatient, outpatient, daycare) and that predictors of treatment outcome may differ among modalities, we specifically focused on outpatient treatments. This systematic review forms part of a larger ongoing study registered in ClinicalTrials.gov (registration ID: NCT05207228) that aims to test the efficacy (dropout and abstinence rates) of a web-based treatment in a sample of CUD outpatients.

### Main outcome variables

The main outcome variables were cocaine abstinence and treatment retention in patients diagnosed with CUD. All RCTs that specifically measured potential predictors of cocaine use in individuals in outpatient treatment were included.

To guide the analysis, we developed the following review question in accordance with the recommendations in the PICO (Population, Intervention/Exposure, Comparison and Outcome) framework for systematic reviews: “What factors predict cocaine dependence and treatment outcomes in adult outpatients with CUD?”. The target population was comprised of adults in outpatient treatment diagnosed with CUD. Given the highly heterogeneous psychosocial and pharmacological interventions for CUD, we did not specify any specific intervention or comparison. We evaluated the following potential predictors of treatment retention and abstinence: sociodemographic; cocaine use patterns; comorbid pathologies; personality traits; neuroimaging, biological and genetic markers; and treatment variables.

### Data extraction

All citations from the databases were exported to Mendeley. Next, we removed duplicate studies and those that could not be located. Next, one of the researchers (APL) screened each citation by title and abstract to identify studies for full review, which were then screened by two authors (APL and NMB) to determine if they met eligibility criteria. If there was any doubt regarding whether a study met the inclusion criteria, the same authors discussed these studies on a case-by-case basis, which were included or not based on a consensus decision. Full-text screening was performed and the data were compiled into an extraction table.

The author APL retrieved the following data for each study: 1) general information (title, author, journal, year); 2) study characteristics (design, objectives/hypothesis, participants, methods, inclusion/exclusion criteria, conditions); 3) participant characteristics (age; gender; sociodemographic data; cocaine use variables; comorbid psychiatric disorders; personality traits; use of other substances; neuroimaging; genetic markers; biological markers); 4) treatment outcomes (retention, dropout, relapse, abstinence after treatment) and type of measurement, which includes time and method of assessment, and measurement instrument; 5) intervention (type of intervention, intervention characteristics [number of sessions, individual/group sessions, duration, and frequency of sessions] and number of post-treatment follow-up sessions), and 6) outcomes (predictors of treatment outcomes of individuals with CUD in outpatient treatment) (Table 1).

**Table 1:** Study details: authors, year, sample size, predictors, criteria, treatment type, outcomes and bias assessed noted

Study ID (Author, year)	N	Predictor variables	Inclusion criteria	Type of treatment	Treatment outcome	Risk of bias (RoB-2)
Ahmadi, J., Kampman, K. & Dackis, C. (2006) [10]	402	CSSA score; urine toxicology screen result	Age between 18 and 65; meet DSM-IV criteria for cocaine dependence; in the past 30 days used no less than 200$ worth of cocaine	Pharmacotherapy (randomized to different medications, including placebo) and individual cognitive-behavioral (CBT) coping skills therapy	Three continuous weeks of abstinence from cocaine were reported by urine drug screening (UDS); a 50% reduction in the ASI composite drug scores at the end of the trial; no self-reported cocaine use during the last for weeks of the trial	Risk of bias arising from the randomization process: high Risk of bias due to deviations from the intended interventions: some concerns Missing outcome data: high Risk of bias in measurement of the outcome: some concerns Risk of bias in selection of the reported result: low
Alessi, S., Rash, C. & Petry, N. (2011) [11]	393	LDA; contingency management (CM)	Being at least 18 years old; past-year cocaine abuse or dependence; able to comprehend study procedures	Psychotherapy: randomized to standard treatment or CM	Long term abstinence; retention, measured by UDS	Risk of bias arising from the randomization process: low Risk of bias due to deviations from the intended interventions: some concerns Missing outcome data: low Risk of bias in measurement of the outcome: some concerns Risk of bias in selection of the reported result: some concerns
Barber, J.P., Luborsky, L., Crits-Christoph, P. et al. (1999) [12]	252	Treatment alliance	DSM-III-R cocaine dependent outpatients who participated in the pilot training phase of the National Institute for Drug Abuse (NIDA) Collaborative Cocaine Treatment Study (CCTS)	Psychotherapy: randomized to individual drug counselling (IDC), supportive-expressive therapy (SE) or cognitive therapy (CT). All participants underwent group drug counselling (GDC)	ASI scores; BDI scores; BSI scores; cocaine use during last week	Risk of bias arising from the randomization process: some concerns Risk of bias due to deviations from the intended interventions: high Missing outcome data: low Risk of bias in measurement of the outcome: some concerns Risk of bias in selection of the reported result: low
Barber, J., Luborsky, L., Gallop, R. et al. (2001) [13]	308	Treatment alliance	Patients aged 18 to 60 years; a principal current diagnosis of cocaine dependence or cocaine dependence in early partial remission (as determined by DSM-IV criteria); cocaine use at least once in the 30 days before enrolment; a current postal address and plan to live in the area for the next 2 years; be able to provide the name of at least 1 person who can generally locate their whereabouts; be able to understand and complete the assessment measures; signature of the informed consent	Psychotherapy: randomized to IDC, SE or CT. All participants underwent GDC	Treatment retention	Risk of bias arising from the randomization process: some concerns Risk of bias due to deviations from the intended interventions: some concerns Missing outcome data: low Risk of bias in measurement of the outcome: some concerns Risk of bias in selection of the reported result: low
Bisaga, A., Aharonovich, E., Cheng, W. et al. (2010) [14]	112	Baseline abstinence; weekly proportion of craving at baseline	Men and women aged between 18 and 60 years old; meet DSM-IV criteria for current cocaine dependence; cocaine use at least 4 days in the previous month; provide a urine sample positive for cocaine metabolites	Pharmacotherapy (randomized to memantine or placebo) and CM plus CBT	Weekly proportion of days of cocaine use; sustained abstinence; proportion of days per week with craving for cocaine; retention in treatment	Risk of bias arising from the randomization process: low Risk of bias due to deviations from the intended interventions: some concerns Missing outcome data: low Risk of bias in measurement of the outcome: low Risk of bias in selection of the reported result: low
Blevins, D., Wang, X., Sharma, S. et al. (2019) [15]	142	Impulsiveness	Be in good physical health (determined by a complete physical examination, electrocardiogram [EKG], and laboratory screening); diagnosis of CUD according to DSM-IV criteria; be randomized into double-blind treatment; meet the criterion of recent history of cocaine use by providing at least one cocaine-positive urine specimen (> 300 ng/ml) during the screening visit or two weeks of baseline	Pharmacotherapy (randomized to topiramate or placebo) and CBT	Proportion of cocaine-free days	Risk of bias arising from the randomization process: low Risk of bias due to deviations from the intended interventions: high Missing outcome data: low Risk of bias in measurement of the outcome: low Risk of bias in selection of the reported result: low
Brewer, J., Worhunsky, P., Carroll, K. et al. (2008) [16]	20	Pre-treatment brain activation	English-speaking adults; meet current DSM-IV criteria for cocaine dependence via structured clinical interviews (SCID)	Data from two RCTs. Pharmacotherapy and psychotherapy Study 1: randomized to CBT + TAU or TAU (*n* = 3) Study 2: randomized to CBT + placebo, CBT + disulfiram, CBT + placebo + CM, CBT + disulfiram + CM	Percent of urine drug screens negative for cocaine; percent days abstinent; and treatment retention	Risk of bias arising from the randomization process: low Risk of bias due to deviations from the intended interventions: low Missing outcome data: low Risk of bias in measurement of the outcome: low Risk of bias in selection of the reported result: low
Carroll, K., Herman, A., Devito, E. et al. (2015) [17]	101	Catechol-O-methyltransferase (COMT) Gene Val158met polymorphism	English-speaking adults; stabilized on methadone (no dose change > 2 months); meet DSM-IV criteria for current cocaine dependence	Pharmacotherapy and psychotherapy: randomized to standard methadone maintenance treatment and computerize CBT or standard treatment alone	Percent days abstinent from cocaine self-report; percent urine specimens testing negative for cocaine metabolites	Risk of bias arising from the randomization process: low Risk of bias due to deviations from the intended interventions: some concerns Missing outcome data: low Risk of bias in measurement of the outcome: low Risk of bias in selection of the reported result: low
Crits-Christoph, P., Gibbons, M., Barber, J. et al. (2007) [18]	487	Acuity of biomedical problems; craving; ARS; expectations for improvement	Age between 18 and 60 years; had to receive a principal diagnosis of cocaine dependence (current or in early partial remission) according to DSM-IV criteria; use of cocaine in the past 30 days	Psychotherapy: randomized to GDC alone, IDC + GDC, SE + GDC or CT + GDC	Sustained abstinence measured by ASI, self-report cocaine inventory, weekly observed urine samples	Risk of bias arising from the randomization process: low Risk of bias due to deviations from the intended interventions: some concerns Missing outcome data: low Risk of bias in measurement of the outcome: some concerns Risk of bias in selection of the reported result: low
Crits-Christoph, P., Johnson, J., Connolly Gibbons, M. et al. (2013) [19]	487	Therapeutic alliance, feedback and advice	Age between 18 and 60 years; cocaine use at least once in the past 30 days; report a stable living situation	Psychotherapy: randomized to GDC alone, IDC + GDC, SE + GDC or CT + GDC	Monthly cocaine use; next session of cocaine use; duration of sustained abstinence	Risk of bias arising from the randomization process: low Risk of bias due to deviations from the intended interventions: low Missing outcome data: low Risk of bias in measurement of the outcome: some concerns Risk of bias in selection of the reported result: some concerns
Crits-Christoph, P., Wadden, S., Gaines, A. et al. (2018) [20]	566	Symptoms of anhedonia	Study 1: achievement of initial engagement in IOP; no psychiatric or medical condition that precluded outpatient treatment; being between 18 and 70 years of age; no IV heroin use within the past 12 months; ability to read at approximately the fourth-grade level; at least a minimum degree of stability in living situation; willingness to participate in research; be randomly assigned to one of the four treatment conditions Study 2: aged between 18 and 60 years; cocaine use at least once in the past 30 days; report a stable living situation	Data from 2 RCT. Psychotherapy: Study 1: (randomized to CM + relapse prevention [RP] or CM) Study 2: randomized to IDC, SE or CT. All participants underwent GDC	During-treatment monthly composite measure of cocaine	Risk of bias arising from the randomization process: low Risk of bias due to deviations from the intended interventions: some concerns Missing outcome data: low Risk of bias in measurement of the outcome: some concerns Risk of bias in selection of the reported result: low
Ehrman, R., Robbins, S. & Cornish, J. (2001) [21]	61	Initial cocaine urine status	DSM-III-R criteria for a diagnosis of cocaine dependence; being enrolled in outpatient treatment for cocaine dependence	Pharmacotherapy (randomized to placebo or ritanserin)	Complete abstinence at four weeks of trial	Risk of bias arising from the randomization process: low Risk of bias due to deviations from the intended interventions: some concerns Missing outcome data: low Risk of bias in measurement of the outcome: low Risk of bias in selection of the reported result: low
Gallop, R., Crits-Christoph, P., Ten Have, T, et al. (2007) [22]	454	Gender	Age between 18 and 60 years; had to receive a principal diagnosis of cocaine dependence (current or in early partial remission) according to DSM-IV criteria; use of cocaine in the past 30 days	Psychotherapy: randomized to GDC alone, IDC + GDC, SE + GDC or CT + GDC	Cocaine abstinence	Risk of bias arising from the randomization process: low Risk of bias due to deviations from the intended interventions: low Missing outcome data: low Risk of bias in measurement of the outcome: low Risk of bias in selection of the reported result: low
Garcia-Fernandez, G., Secades-Villa, R., Garcia-Rodriguez, O. et al. (2011) [23]	50	Abstinence at 1 month of treatment	Being at least 20 years old; meet DSM-IV criteria for active cocaine dependence	CM: randomized to community reinforcement approach (CRA) or to TAU	Abstinence at six months of treatment	Risk of bias arising from the randomization process: some concerns Risk of bias due to deviations from the intended interventions: high Missing outcome data: high Risk of bias in measurement of the outcome: low Risk of bias in selection of the reported result: some concerns
Johnson, J. E., Connolly-Gibbons, M. B. & Crits-Christoph, P. (2011) [24]	487	Gender and race	Age between 18 and 60; diagnosis of cocaine dependence; cocaine as the primary drug; reported cocaine use in the past 30 days	Psychotherapy: randomized to GDC alone, IDC + GDC, SE + GDC or CT + GDC	Self-reported days using cocaine each month during the 6-month treatment period	Risk of bias arising from the randomization process: low Risk of bias due to deviations from the intended interventions: low Missing outcome data: low Risk of bias in measurement of the outcome: some concerns Risk of bias in selection of the reported result: low
Kampman, K., Volpicelli, J., Mulvaney, F. et al. (2002) [25]	76	Urine toxicology screen; CSSA score at baseline	Aged between 18 and 60; admitted for one of four open-label screening medication trials for cocaine dependence; subjects had all used at least US$100 worth of cocaine in the 30 days prior to entering the treatment trial	Pharmacotherapy (randomized to different medications) and CBT	Three weeks of continuous abstinence, measured by UDS	Risk of bias arising from the randomization process: high Risk of bias due to deviations from the intended interventions: high Missing outcome data: low Risk of bias in measurement of the outcome: low Risk of bias in selection of the reported result: some concerns
McKay, J., Alterman, A., Cacciola, J. et al. (2000) [26]	127	Antisocial Personality Disorder	DSM-III-R diagnoses of cocaine dependence (lifetime); cocaine use in the prior 6 months	Psychotherapy: randomized to standard group or individualized RP	Percent days of cocaine use; percent days of heavy alcohol use; percent days totally abstinent	Risk of bias arising from the randomization process: low Risk of bias due to deviations from the intended interventions: some concerns Missing outcome data: low Risk of bias in measurement of the outcome: low Risk of bias in selection of the reported result: some concerns
McKay, J., Merikle, E., Mulvaney, F. et al. (2001) [27]	132	Current cocaine use	DSM-III-R diagnoses of cocaine dependence (lifetime); cocaine use in the previous 6 months	12-Step focused group treatment vs. individualized relapse prevention continuing care	Percentage of days of cocaine use and frequency of use	Risk of bias arising from the randomization process: some concerns Risk of bias due to deviations from the intended interventions: high Missing outcome data: low Risk of bias in measurement of the outcome: some concerns Risk of bias in selection of the reported result: low
McKay, J., Van Horn, D., Rennert, L. et al. (2013) [28]	766	Age; education; cocaine and alcohol use at baseline; self-efficacy; commitment to abstinence; social support; depression; other problem severity measures; self-help participation; self-help beliefs; readiness to change	Studies 1 and 2: meet criteria for current DSM- IV cocaine dependence at the time of entrance to treatment Study 3: meet lifetime criteria for cocaine dependence; cocaine use in the six months prior to entrance to treatment; willingness to participate in research; be randomly assigned to one of the three continuing care conditions in each study; no psychiatric or medical condition that precluded outpatient treatment; age between 18 and 65; no IV heroin use within the past 12 months; ability to read at approximately the 4th grade level; at least a minimum degree of stability in living situation	Data from 3 RCT. Psychotherapy: Study 1: randomized to TAU or CBT/RP or telephone continuing care Study 2: TAU or extended telephone monitoring only or extended telephone monitoring counseling (TMC) Study 3: TAU or TMC or TMC + incentives for attendance	Cocaine use transitions	Risk of bias arising from the randomization process: low Risk of bias due to deviations from the intended interventions: some concerns Missing outcome data: low Risk of bias in measurement of the outcome: low Risk of bias in selection of the reported result: some concerns
Moeller, F., Dougherty, D., Barrat, E. et al. (2001) [29]	41	Baseline impulsivity	Presence of current cocaine dependence by DSM-IV criteria; lack of current or past DSM-IV Axis I diagnosis other than substance dependence or substance induced mood disorder; willingness to complete questionnaires	Pharmacotherapy (randomized to buspirone or placebo) and relapse prevention group therapy	Dropout	Risk of bias arising from the randomization process: low Risk of bias due to deviations from the intended interventions: high Missing outcome data: high Risk of bias in measurement of the outcome: low Risk of bias in selection of the reported result: some concerns
Nuijten, M., Blanken, P., Van Den Brink, W., et al. (2016) [30]	65	Baseline impulsivity; baseline response inhibition; baseline cognitive interference; baseline attentional bias	Age 18 years or older; cocaine dependent according to DSM-IV criteria; cocaine use by means of basing (crack-cocaine) for at least 8 days in the previous month	Pharmacotherapy and psychotherapy: randomized to CBT + modafinil or CBT alone	Treatment retention; self-reported crack-cocaine use days within the 30 days preceding the assessment; change in self-reported crack-cocaine use days; changes in response inhibition, cognitive interference and attentional bias	Risk of bias arising from the randomization process: high Risk of bias due to deviations from the intended interventions: some concerns Missing outcome data: high Risk of bias in measurement of the outcome: low Risk of bias in selection of the reported result: some concerns
Rash, C., Alessi, S. & Petry, N. (2008) [31]	393	Years of cocaine use; centered long duration abstinence (LDA)	Aged 18 years or older; presence of past year cocaine abuse or dependence according to DSM-IV criteria	Psychotherapy: randomized to standard care or standard care + CM)	Treatment retention; longest duration of objectively-verified continuous abstinence achieved; proportion of negative samples submitted	Risk of bias arising from the randomization process: some concerns Risk of bias due to deviations from the intended interventions: some concerns Missing outcome data: low Risk of bias in measurement of the outcome: low Risk of bias in selection of the reported result: some concerns
Rash, C., Andrade, L. & Petry, N. (2013) [32]	418	Type of income and treatment condition	Being an adult; English-speaking; cocaine dependent patients initiating outpatient substance abuse treatment between 2003 and 2007	Psychotherapy: randomized to standard treatment (ST), ST + abstinence-based CM ($250 average maximum available), ST + attendance-based CM ($250 average maximum available), ST + abstinence-based CM ($560 average maximum available)	LDA	Risk of bias arising from the randomization process: low Risk of bias due to deviations from the intended interventions: some concerns Missing outcome data: low Risk of bias in measurement of the outcome: low Risk of bias in selection of the reported result: some concerns
Rash, C., Burki, M., Montezuma-Rusca, J. et al. (2016) [33]	493	Treatment condition; education	Age 18 years or older; beginning intensive outpatient treatment at a substance abuse treatment clinic; ability to understand study procedures; DSM-IV substance use diagnosis	Psychotherapy: randomized to SC or CM	Retention in treatment; LDA; and the percentage of samples submitted that tested negative during treatment	Risk of bias arising from the randomization process: low Risk of bias due to deviations from the intended interventions: high Missing outcome data: low Risk of bias in measurement of the outcome: low Risk of bias in selection of the reported result: some concerns
Schmitz, J., Mooney, M., Green, C. et al. (2009) [34]	75	Impulsivity	Cocaine dependence, but no other Axis I disorders	Pharmacotherapy and psychotherapy: Randomized to CBT-abstinence based CM (behavioral therapy) + citalopram or behavioral therapy + placebo	Treatment retention	Risk of bias arising from the randomization process: low Risk of bias due to deviations from the intended interventions: low Missing outcome data: low Risk of bias in measurement of the outcome: low Risk of bias in selection of the reported result: some concerns
Secades-Villa, R., García-Fernández, G., Peña-Suárez, E. et al. (2013) [35]	118	Treatment condition; EuropASI psychiatric composite scores	Being at least 20 years old; meet DSM-IV criteria for active cocaine dependence; not presenting serious psychopathological disorders or active opioid dependence	Psychotherapy: randomized to two different CM conditions (CRA or CRA + voucher)	Treatment retention and the duration of objectively verified continuous cocaine abstinence	Risk of bias arising from the randomization process: some concerns Risk of bias due to deviations from the intended interventions: high Missing outcome data: high Risk of bias in measurement of the outcome: low Risk of bias in selection of the reported result: some concerns
Siqueland, L., Crits-Christoph, P., Frank, A. et al. (1998) [36]	286	Basic demographic variables; measures of current and past drug use severity; psychiatric comorbidity	Primary current cocaine dependence; use of cocaine in the last 30 days; stable living situation	Psychotherapy: randomized to IDC, SE or CT. All participants underwent GDC	Dropout	Risk of bias arising from the randomization process: low Risk of bias due to deviations from the intended interventions: low Missing outcome data: low Risk of bias in measurement of the outcome: low Risk of bias in selection of the reported result: low
Siqueland, L., Crits-Christoph, P., Gallop, R. et al. (2002) [37]	487	Age; race; employment status; education; mode of cocaine use; treatment condition; psychiatric severity	Cocaine use in the past 30 days; principal diagnosis DSM-IV cocaine dependence (current or in early partial remission); ages between 18 and 60	Psychotherapy: randomized to GDC alone, IDC + GDC, SE + GDC or CT + GDC	Retention in treatment	Risk of bias arising from the randomization process: low Risk of bias due to deviations from the intended interventions: low Missing outcome data: low Risk of bias in measurement of the outcome: some concerns Risk of bias in selection of the reported result: low
Stulz, N., Thase, M., Gallop, R. et al. (2011) [38]	487	Depressive symptoms	Principal diagnosis of cocaine dependence according to DSM- IV; cocaine use during the past 30 days	Psychotherapy: randomized to GDC alone, IDC + GDC, SE + GDC or CT + GDC	Next month drug use severity	Risk of bias arising from the randomization process: low Risk of bias due to deviations from the intended interventions: low Missing outcome data: low Risk of bias in measurement of the outcome: some concerns Risk of bias in selection of the reported result: low
Turner, T., LaRowe, S., Horner, M. et al. (2009) [39]	84	Percent of perseverative errors from the WCST	Be able to give informed consent by the University Institutional Review Board; meet the DSM-IV criteria for cocaine dependence; age between 21 and 50 years; had used cocaine via smoked or intravenous route; unable to cease cocaine use for at least 3 weeks during the 90 days prior to entry in the study; used a minimum of $1,000 worth of cocaine in the 90 days prior to enrolment; had used cocaine three time per week in the month prior to induction into the study	Pharmacotherapy and psychotherapy: randomized to amlodipine + CBT or placebo + CBT	Treatment retention; total number of negative urine drug screens	Risk of bias arising from the randomization process: some concerns Risk of bias due to deviations from the intended interventions: low Missing outcome data: low Risk of bias in measurement of the outcome: low Risk of bias in selection of the reported result: low
Winhusen, T., Theobald, J. & Lewis, D. (2019) [40]	290	Baseline sleep disturbance	Cigarette smokers interested in quitting smoking and enrolled in outpatient SUD treatment for stimulant dependence; be in good physical health; not currently being treated for nicotine dependence; no medical or psychiatric conditions that would make study participation unsafe	Pharmacotherapy and psychotherapy (randomized to treatment as usual [TAU] or TAU + smoking cessation treatment)	Cocaine abstinence and self-report of no cocaine use	Risk of bias arising from the randomization process: low Risk of bias due to deviations from the intended interventions: some concerns Missing outcome data: low Risk of bias in measurement of the outcome: low Risk of bias in selection of the reported result: low
Wong, C., Anthony, S., Sigmon, S. et al. (2004) [41]	126	Early abstinence	Be at least 18 years old; meet criteria for cocaine dependence according to DSM–III–R; had to have used cocaine within the 30 days prior to the intake interview	Psychotherapy randomized to different CM conditions (CRA + contingent vouchers or CRA + non-contingent vouchers, vouchers only)	Coping self-efficacy; abstinence	Risk of bias arising from the randomization process: some concerns Risk of bias due to deviations from the intended interventions: high Missing outcome data: low Risk of bias in measurement of the outcome: some concerns Risk of bias in selection of the reported result: some concerns

### Assessment of risk of bias

One researcher (APL) assessed the risk of bias in the individual studies using the revised Cochrane risk-of-bias tool for randomized trials (RoB-2), which includes five different domains: randomization process, deviations from intended interventions, missing outcome data, measurement of the outcome, and selection of the reported result [42]. The Risk of bias synthesis can be seen in Table 1.

## Results

### Data synthesis strategy

The results are presented as a narrative synthesis. A PRISMA flow-chart was prepared to illustrate the selection process of the RCTs included. The search yielded a total of 566 records; of these, 32 met the selection criteria and were included in the systematic review (Fig. 1 and additional file 4). Next, the data in the extraction table were summarized to perform a qualitative synthesis and to organize the information into sections.

A metanalytic synthesis was not performed, mainly due to the heterogeneity (interventions, treatment duration, follow-up period, outcome variables) of the studies included in this systematic review. Given the wide variability in the interventions performed, it was not possible to unify all of the studies under a single intervention variable. In addition, these studies included numerous other potential predictors of treatment outcomes (treatment duration, follow-up period, and outcome variables), which were also heterogenous among these studies. In short, due to the clinical and methodological heterogeneity, a metanalytic synthesis would have been an inappropriate study design.

### Description of studies

After completion of the data extraction process, 32 RCTs (or secondary analyses of data from an RCT) were included in the review. The patients in those 32 RCTs were randomized to a wide range of different treatment conditions, either pharmacological or psychotherapeutic. As a result, the review includes information about different potential predictors of treatment outcomes in cocaine users, which are described below in separate sections by categories, as follows: sociodemographic variables; cocaine use variables; comorbid psychiatric disorders; personality traits; neuroimaging; genetic markers; and biological markers (Table 1).

Numerous variables were evaluated in these RCTs as possible predictors for CUD, which also assessed the association between the variable and treatment outcomes (Table 2).

**Table 2:** Authors, years, predictors and outcomes found for each record in the systematic review

Study ID	Predictor of treatment outcome	Outcome
*Sociodemographic variables*		
McKay et al. (2013) Siqueland et al. (1998, 2022) [28, 36, 37]	Age	Younger age predicts higher relapse and dropout
Gallop et al. (2007) Johnson et al. (2011) [22, 24]	Gender	No effect
Johnson et al. (2011) Siqueland et al. (2002) [24, 37]	Race	Ethnic minority predicts a shorter treatment retention
McKay et al. (2013) Rash et al. (2013, 2016) Siqueland et al. (1998, 2002) [28, 32, 33, 36, 37]	Education and employment status	Mixed findings
Rash et al. (2013) [32]	Type of income	Income from public assistance predicts greater longest duration abstinence (LDA), while illegal income is associated with shorter LDA
*Cocaine use variables*		
McKay et al. (2001, 2013) Rash et al. (2008) Siqueland et al. (1998) [27, 28, 31, 36]	Years of cocaine use and current cocaine use	Fewer years of cocaine use and less cocaine use in the previous 30 days predict higher abstinence and treatment retention, and fewer days of use
Siqueland et al. (2002) [37]	Mode of cocaine use	Crack smokers and intravenous users remained in treatment for a shorter period of time
Siqueland et al. (1998, 2002) [36, 37]	Severity of the addiction	No effect
Ahmadi et al. (2006) Kampman et al. (2002) [10, 25]	Cocaine withdrawal symptoms	Fewer cocaine withdrawal symptoms predict higher abstinence, lower ASI scores and no self-reported cocaine use in the last weeks
Ahmadi et al. (2006) Ehrman et al. (2001) Kampman et al. (2002) Rash et al. (2013) [10, 21, 33,25 ]	Urine toxicology screen	A negative urine sample predicts greater abstinence, a decrease in severity, and no-self reported cocaine use at treatment end
Bisaga et al. (2010) Garcia-Fernandez et al. (2011) Wong et al. (2004) [14, 23, 41]	Baseline abstinence and LDA during treatment	Baseline abstinence and LDA during treatment predict long-term abstinence
Bisaga et al. (2010) Crits-Christoph et al. (2007) [14, 18]	Craving	Higher craving predicts less abstinence and higher craving during treatment
*Comorbid conditions*		
Crits-Christoph et al. (2018) McKay et al. (2013) Secades-Villa et al. (2013) Siqueland et al. (1998, 2002) Stulz et al. (2011) [20, 28, 35–38]	Anhedonia, depressive symptoms and psychiatric severity	Mixed findings
Winhusen et al. (2019) [40]	Baseline sleep disturbance	No effect
McKay et al. (2000) [26] Siqueland et al. (2002) [37]	Antisocial personality disorder	No effect
*Personality traits*		
Blevins et al. (2019) [15] Moeller et al. (2001) [29] Nuijten et al. (2016) [30]	Impulsivity	Mixed findings. Although some evidence shows that not all the BIS-11 sub-scales predict CUD treatment outcomes, the preponderance of evidence suggests that greater impulsivity predicts greater addiction severity and withdrawal symptoms, and a shorter period of time in treatment and a greater use of cocaine in the month prior to treatment
*Neurocognitive functioning*		
Nuijten et al. (2016) [30]	Baseline response inhibition, cognitive interference, and attentional bias	Good response inhibition, low cognitive interference and less attentional bias predict fewer days of crack-cocaine use
Turner et al. (2009) [39]	Cognitive flexibility and problem solving	More mistakes on a problem-solving task predict lower treatment retention
*Neuroimaging*		
Brewer et al. (2008) [16]	Brain activation	Better performance on the Stroop Task predicts greater treatment retention and abstinence
*Genetic markers*		
Carroll et al. (2015) [17]	Catechol-O-methyltransferase Gene Val158met polymorphism	Polymorphism Val158met of the COMT gene predicts greater reduction in cocaine use
*Treatment features*		
Alessi et al. (2011) Rash et al. (2013, 2016) Secades-Villa et al. (2013) Siqueland et al. (2002) [11, 32, 33, 35, 37]	Treatment condition	Mixed findings; however participants who undergo CM have a better prognosis
Barber et al. (1999, 2001) Crits-Christoph et al. (2013) [12, 13, 19]	Treatment alliance and advice giving	Mixed findings; however, a greater use of advice giving predicts lower abstinence
Crits-Christoph et al. (2007) McKay et al. (2013) [18, 28]	Expectations for improvement and commitment to abstinence	A greater engagement with treatment predicts greater odds of abstinence
Crits-Christoph et al. (2007) [18]	Acuity for biomedical problems	Greater acuity for biomedical problems predicts sustained abstinence
McKay et al. (2013) [28]	Self-help beliefs, self-help participation and self-efficacy	Greater self-help beliefs, self-help participation and self-efficacy predict switching from cocaine use to abstinence

### Narrative synthesis

#### Sociodemographic variables: age, gender, ethnicity, education and employment status and type of income

Three RCTs found that age was a significant predictor of treatment retention, with younger patients less likely to remain in treatment and more likely to drop out earlier [28, 36, 37]. Even among patients who had completed the stabilization phase, younger patients were more likely to drop out than older patients. Moreover, younger patients randomized to a specific treatment dropped out earlier in the treatment process than older patients [36, 37]. One trial found that older age was a predictor of sustained cocaine abstinence or, among current users, of transitioning to abstinence [28]. The findings of those trials suggest that it may be possible to reduce the likelihood of treatment dropout by identifying and addressing the concerns of younger patients through prevention campaigns.

Two RCTs found that gender was not a predictor of cocaine use at six months posttreatment [22, 24]. Interestingly, [22] observed significant differences between genders in the transition from abstinence to cocaine use, with men transitioning nearly two times as fast as women. In other words, women who use cocaine are more likely to keep using it while women who are abstinent are more likely to remain abstinent. By contrast, men who are abstinent are at higher risk of switching back to cocaine use and vice versa [22]. However, the low proportion of women in both studies (23% in each RCT) could have at least partially influenced these findings, in part by reducing the studies’ power to identify gender as a predictor of treatment outcomes in CUD [22, 24].

Two RCTs reported that ethnicity was a predictor of treatment retention, finding that ethnic minorities tend to remain in treatment during less time and drop out sooner [24, 37]. Interestingly, [37] found that African-American participants living alone remained in treatment longer than those living with a partner or spouse; by contrast, the opposite was true for American Caucasians. Although no data on cocaine use among the patients’ partners was collected, many African-American participants reported difficulties in achieving abstinence or continuing with treatment because people close to them continued using drugs [37]. In their RCT, [24] found that, during treatment for CUD, African-American women had lower rates of past self-disclosure, a lower percent of time at talk, less receipt of advice, and less non-positive feedback that non-Hispanic white women [24]. These data suggest that African-American women, the most vulnerable group, should receive more attention in treatment programs to enhance their motivation to change.

Five of the RCTs in this review found that education and employment status were significant predictors of treatment retention and longest duration of abstinence (LDA) during treatment. In other words, less educated and/or unemployed participants remained in treatment for a shorter period of time, and lower educational levels were associated with a shorter duration of abstinence [28, 32, 33, 36, 37]. Unemployed men remained in treatment longer than unemployed women (82 vs. 56 days), while employed women had higher treatment retention rates than employed men (148 vs. 103 days). These findings suggest that unemployed and less educated women, who are the most vulnerable group, might require treatment interventions that target other psychosocial needs, such as financial concerns or job search skills, in order to increase treatment retention [37]. Only one trial [28] found that a lower educational level predicted continued abstinence or transition to cocaine-free status, with a negative correlation between years of education and transitioning to cocaine abstinence. Based on the mixed evidence in these trials, the role of education and employment status as predictors of treatment retention remains to be clarified.

The role of income received during treatment has received scant attention as a potential predictor of treatment outcomes in CUD. To date, only one study [32] has included this variable as a potential predictor. After controlling for demographic and baseline characteristics, the authors of that study found that income from illegal activities and public assistance were significant predictors of LDA. However, income from public assistance sources was associated with greater LDA whereas illegal income was associated with a shorter LDA [32].

#### Cocaine use variables: years of cocaine use and current use; mode of use, addiction severity index, cocaine withdrawal symptoms, toxicology screening and duration of abstinence, and craving

Four RCTs found that years of cocaine use, LDA, cocaine use in the 30 days prior to treatment entrance, and current cocaine use were strong predictors of treatment retention and abstinence (based on urine drug tests). Less cocaine use in the 30 days prior to treatment entrance, fewer years of cocaine use, and a greater LDA were all predictive of higher abstinence and treatment retention rates [28, 31, 36]; these same variables were also predictors of a lower frequency and proportion of days of cocaine use [27]. In one study, each additional year of cocaine use decreased the odds of a negative urine drug sample at the 9-month follow-up by 5% [31]. These findings indicate that years of cocaine use and current cocaine use status are robust predictors of treatment outcomes in cocaine users.

The mode of cocaine use has not been widely studied as a predictor of treatment outcomes in cocaine users. However, one RCT [37] found that the mode of use predicted treatment retention. Crack and intravenous cocaine users remained in outpatient treatment fewer days than intranasal users (88 vs. 134 days, respectively), which suggested that crack smokers and intravenous users have a worse prognosis than intranasal users.

Two RCTs found that addiction severity, measured by the Addiction Severity Index (ASI), a tool used to assess the impact of alcohol and drug use on seven potential problem areas (medical, employment/support status, alcohol, drug, legal, family/social and psychiatric) does not predict time in treatment nor time to dropout among patients receiving treatment after completing the stabilization phase [36, 37]. An important finding of that RCT was that the heaviest users spent the same time in treatment as user with less severe addictions [37]. These findings suggest that many of the participants in those studies were not well-suited for outpatient treatment or not yet ready to change; in addition, the heaviest users (those with more days of cocaine use in the previous month) were less likely to complete the stabilization phase and thus less likely to be randomized to treatment [36].

Two RCTs found that lower scores on the CSSA, a tool used to measure cocaine withdrawal symptoms, was a significant predictor of three weeks of continuous abstinence, a 50% reduction in the ASI composite drug scores at the end of treatment, and no self-reported cocaine use during the last four weeks [10, 25]. More specifically, subjects with CSSA scores > 21 were twelve times more likely to fail to reach three continuous weeks of abstinence [25]. These results suggest that psychological treatments that target CUD should emphasize coping strategies to help patients better manage withdrawal symptoms, thereby limiting the potential impact of these symptoms on treatment outcomes, which would likely improve prognosis.

Four RCTs found that a negative urine sample predicted three continuous weeks of abstinence, a 50% reduction in drug problem severity, and no self-reported cocaine use at the end of treatment [10, 21, 25, 32]. This variable was also a significant predictor of long-term abstinence (up to 6 months after treatment completion) [14, 23, 41].

In one study [14], patients who had achieved abstinence at baseline had 70% fewer days of cocaine use compared to patients who were not abstinent at baseline. In addition, patients who were abstinent at baseline but later dropped out of treatment were more likely to become abstinent again at a later time point. Furthermore, patients who achieved abstinence after one month of treatment were 14 times more likely than those who were still using at that time point to remain abstinent at the 6-month follow-up [23].

The predictive capacity of a negative urine test was stronger when combined with cocaine withdrawal symptoms (measured by the CSSA). More specifically, a negative urine drug test combined with lower scores on the CSSA was the best predictor for ≥ 3 continuous weeks of abstinence, a 50% reduction in drug problem severity, and no self-reported cocaine use at the end of treatment [10, 21, 25]. Moreover, a single positive urine test at treatment entry was a significant predictor of non-abstinence at the end of treatment [21].

Two RCTs found that the LDA (consecutive weeks of negative urine samples during treatment) predicted abstinence at 9-months posttreatment. In addition, the greater the number of negative samples submitted during treatment, the higher the long-term abstinence rate [11, 31]. Specifically, [31] and colleagues found that every one week increase in LDA increased the odds of a negative urine test by 21%.

Two RCTs found that baseline craving levels (measured by the CSSA) predicted abstinence and craving intensity during treatment. That is, higher levels of craving at baseline predicted fewer months of consecutive abstinence. In addition, a higher proportion of days per week of craving before the start of treatment predicted a higher craving proportion during treatment [14, 18].

#### Comorbid conditions: anhedonia, depressive symptoms and psychiatric severity; sleep disturbance, and antisocial personality disorder

Findings regarding the predictive capacity of psychiatric symptoms have been mixed. One RCT showed that self-reported anhedonia symptoms (from Beck’s Depression Inventory [BDI]) were strong predictors of poor treatment response, with higher scores in anhedonia symptoms predicting a worse prognosis [20]. However, when anhedonia symptoms were excluded from the BDI, the total score was not predictive of treatment outcomes [20], which is in line with the finding reported in another RCT, in which psychiatric severity alone was not a predictor of treatment dropout [37]. Nevertheless, four RCTs found that psychiatric symptoms (as measured by the European version of the ASI, EuropASI) and depressive symptoms predicted abstinence and treatment adherence, with more severe psychiatric and depressive symptoms indicating shorter periods of cocaine abstinence and poorer treatment adherence [28, 35, 36, 38]. Importantly, one RCT found that even though participants with depression or depressive symptoms had lower rates of treatment adherence, when these patients did adhere to treatment, they were usually more motivated to continue treatment to alleviate symptoms associated with depression and cocaine use [36].

The role of sleep disturbance as a possible predictor of treatment outcomes in cocaine users is not well-understood, mainly because only limited data are available. However, [40] (a secondary analysis of data from a multi-site RCT) found that, contrary to the initial hypothesis, baseline sleep disturbance were not significant predictors of end-of-treatment abstinence. However, the presence of a sleep disturbance was a significant predictor of three mediators: cocaine craving, anxiety, and depression, which in turn were predictors of low rates of end-of-treatment abstinence [40].

Two RCTs compared patients with and without a diagnosis of antisocial personality disorder (APD) to determine the predictive capacity of this variable. However, APD did not predict differential response to outpatient continuing care treatment. Similarly, APD was not a predictor of relapse or treatment retention among cocaine users [26, 37]. Nevertheless, [26] found that patients with APD had significantly worse medical and psychiatric problems than non-APD patients at the beginning of outpatient continuing care and during follow-up.

#### Personality traits: impulsivity

Impulsivity plays an important role in substance use disorders, including CUD, and several studies have found that baseline impulsivity is a robust predictor of treatment outcomes. Three of the RCTs included in this review found that baseline impulsivity, measured with the BIS-11, predicted cocaine use, treatment retention, and severity of use and withdrawal symptoms. Compared to low baseline impulsivity levels, high levels of impulsivity at baseline predicted more severe addiction and withdrawal symptoms, a shorter period of time in treatment (i.e., earlier dropout), and a significantly greater cocaine use within the last 30 days of treatment [15, 29, 30]. Schmitz and colleagues performed a secondary analysis of data from [29] and showed that higher scores on indices of non-planning impulsivity predicted ≥ 2 weeks of abstinence; by contrast, indices of the attentional, motor, and inhibitory-control components of impulsivity were not significant predictors of treatment retention [34].

#### Neurocognitive functioning: baseline response inhibition, cognitive interference, attentional bias; cognitive flexibility and problem solving

The impact of neurocognitive variables on treatment outcomes in cocaine users has received scant attention. An RCT was conducted to evaluate baseline response inhibition, cognitive interference, and attentional bias as possible predictors of treatment retention and crack cocaine use. Those authors found that good response inhibition, low baseline cognitive interference, and low baseline attentional bias did not predict the number of CBT sessions attended. By contrast, those variables predicted fewer days of crack cocaine use during the last 30 days of treatment [30].

One trial evaluated cognitive flexibility and problem solving as potential predictors of treatment outcomes. In that trial, a high percentage of perseverative errors on the Wisconsin Card Sorting Test (WCST), the most widely used tool to assess cognitive flexibility and problem solving, was a robust predictor of treatment dropout. In other words, patients who repeated mistakes on a problem-solving task discontinued treatment earlier than patients who performed better on the WCST [39].

#### Neuroimaging: brain activation

Brewer and colleagues [16] evaluated brain activation, measured by functional magnetic resonance imaging (fMRI) during performance of the Stroop Task as a potential predictor of treatment outcomes in cocaine users. In that trial, better performance on colour naming and interference predicted greater treatment retention. With regards to cognitive control and behavioral therapy for cocaine use, the results of that trial showed that activation in specific cortico-striatal regions during the Stroop Task was associated with reported abstinence and cocaine-free urine tests. In addition, activation of the dorsolateral prefrontal cortex (dlPFC) was inversely correlated with treatment retention; participants with lower dlPFC activation remained in treatment for a longer period of time. These findings suggest that brain activation might be a more sensitive measure for predicting treatment outcomes.

#### Genetic markers: catechol-O-methyltransferase (COMT) Gene Val158met polymorphism

Studies on genetic markers as potential predictors of treatment outcomes in CUD are limited. In fact, only one RCT [17] has been conducted to explore the role of the COMT Gene Val158met polymorphism as a possible predictor of treatment outcomes in cocaine users. COMT is a regulator of catecholamines in the brain, and the COMT gene polymorphism (Val158met) predicted greater reductions in cocaine use over time. When subjects with the Val allele were assigned to a web-based, computerized CBT treatment program, they were more likely to achieve ≥ 3 weeks of continuous abstinence and to present a higher percentage of days of abstinence during treatment compared to patients who carried the Met/Met allele [17].

#### Treatment features: treatment condition; therapeutic alliance and advice giving; expectations for improvement and commitment to abstinence, and acuity for biomedical problems

The treatment condition has emerged as a robust predictor of treatment outcomes, with four different trials finding that contingency management is predictive of long-term abstinence, higher treatment retention rates, and a higher proportion of negative urine samples [11, 32, 33, 35]. One RCT found that CM was especially beneficial in terms of treatment retention in cocaine users who also used marijuana because these patients tend to drop out of treatment relatively quickly without CM [11]. Another RCT found that treatment outcomes were better in patients who received individual and/or group drug counselling compared to patients randomized to other treatments, such as cognitive therapy or supportive-expressive therapy (a psychodynamic approach) [37].

Two RCTs found that stronger therapeutic alliance is not predictive of cocaine use (ASI) at 6 months posttreatment [12, 13]. However, therapeutic alliance does appear to predict cocaine use at one month posttreatment and also improves depressive symptoms (as measured by the BDI) in patients who remain in treatment versus those who discontinue treatment earlier in the process [12]. Moreover, therapeutic alliance can predict retention across various treatment conditions. In patients who received supportive-expressive therapy or individual drug counselling, a stronger alliance predicted a longer period of time in treatment [13]. Another RCT showed that weak therapeutic alliance in patients receiving group drug counselling was a significant predictor of higher drug use (measured by urinalyses and self-report measures) at the next treatment session, and lower treatment retention rates [19].

In the trial performed by Crits-Chrisoph and colleagues, [19] advice giving, whether from other patients or from the counsellor, predicted abstinence and cocaine use. More specifically, a greater use of advice predicted fewer months of abstinence and more days of cocaine use, including next session of cocaine use.

Several other variables can predict cocaine abstinence. Two RCTs found that expectations for improvement and commitment to abstinence were both strong predictors of sustained abstinence. In other words, a higher level of treatment engagement increases the odds of achieving abstinence [18, 28]. According to Crits-Christoph and colleagues [18], the mechanism underlying the association between higher engagement and better outcomes is probably that expectations for improvement increase therapeutic alliance, which is associated with better treatment outcomes. McKay and colleagues [28] found that self-help beliefs, self-help participation, and self-efficacy also play an important role in transitioning from cocaine use to abstinence. Thus, higher levels of those three variables predicted the transition from cocaine use to abstinence. This finding underscores the key role of expectations on improvement and self-efficacy.

One RCT [18] found that concerns about biomedical problems can also impact the course of the addiction. In that trial, a greater acuity for biomedical problems predicted sustained abstinence. In other words, patients who were more concerned about their own biomedical issues were more likely to achieve sustained abstinence [18].

## Discussion

Cocaine use disorder is a highly complex condition involving the convergence of numerous variables that modulate the addiction prognosis. Our findings show that three variables—younger age, more years of cocaine use, and more frequent cocaine use in the previous 30 days—were significant predictors of relapse and treatment dropout [28, 36, 37]. Regarding the first variable, although it is still unclear why younger age is a predictor of treatment dropout, the lower likelihood of younger patients maintaining intake appointments could be explained by various factors. These factors may include extensive research monitoring requirements and a lack of community-based efforts to inform these patients about other treatment alternatives [36]. From the neurobiological perspective, during adolescence, the brain is still under development, especially the prefrontal cortical regions responsible for emotion regulation and adult-level judgement. Consequently, impulsivity increases, placing youths at greater risk of engaging in drug and other risky behaviors [43, 44]. Referring to more years of cocaine use, the longer the duration of cocaine use, the higher the resistance to change. Moreover, the odds of submitting a negative long-term urine sample decreases with every year of cocaine use [31]. Individuals with a long history of cocaine use should receive a differentiated and more intensive treatment protocol, regardless of other severity variables, such as current cocaine use. Finally, greater cocaine use in the previous 30 days at the 18-month follow-up emerges as a significant predictor of subsequent cocaine use, with no other treatment-related factor or social functioning variable showing significant predictive power for subsequent cocaine use. This finding suggests a temporal progression in which factors related to cocaine dependence treatment play a more relevant role at the beginning of treatment, while those related to social functioning, unrelated to treatment, become more important during the follow-up phase [27].

In terms of gender, none of the reviewed studies found gender to be a significant predictor of CUD treatment outcomes, which aligns with the existing literature on this topic [45]. Nevertheless, it is worth highlighting the importance of considering gender-specific variables when approaching treatment and incorporating them into the strategies for addressing specific vulnerable groups. For instance, women who are victims of gender-based violence present a greater risk of engaging in substance use behaviors [46]. Higher baseline craving appears to be predictive of relapse in CUD, but only limited data are available [7, 14, 18]. However, it is important to note that craving can have a different impact depending on the setting where it appears. When craving occurs in hospitalization settings its management do not turn out so complex since there are more available resources to face it (e.g. immediate care from nursing staff, availability of pharmacological options to address craving, etc.). In contrast, when craving occurs in outpatient settings the patient needs to be more trained in accessing craving management abilities and strategies to prevent relapse, which is a more probable outcome due to the lack of immediate resources. Despite this, craving is a widely observable component in real-life clinical practice that predicts worse CUD treatment outcomes. In this regard, it would be interesting to further study the effects of craving on treatment outcomes in CUD in order to better understand the role of this variable and, if appropriate, to specifically target it in psychosocial treatments within outpatient settings. By contrast, fewer withdrawal symptoms predict less cocaine use severity (lower ASI scores) and no self-reported cocaine use in the previous weeks [10, 25], as well as longer abstinence at baseline does [14, 23, 41]. In fact, one study emphasizes the importance of longer abstinence at baseline, indicating that participants who achieved abstinence after one month of treatment were over 14 times more likely to remain abstinent after six months post-treatment than those who used cocaine four weeks after treatment [23]. In terms of self-efficacy levels higher scores were also predictors of continued and long-term abstinence [28]. In spite of this, studies on the impact of self-efficacy on addictions treatment are scant; however, the trial conducted by McKay and colleagues [28] demonstrates how focusing on specific treatments and objectives can be highly effective, thus providing a model for future studies.

The available literature shows that greater impulsivity (measured by the total score of BIS-11) is predictive of more severe addiction and withdrawal symptoms, earlier treatment discontinuation, and greater cocaine use in the month prior to treatment initiation [15, 29, 30], which is aligned with the current evidence [8]. However, in the study conducted by Schmitz and colleagues, [34] the non-planning impulsivity index only predicted two weeks of abstinence, which suggests that the significance of these findings should be considered cautiously. In terms of the presence of concomitant psychopathology, higher scores on scales measuring depressive symptoms are associated with a worse prognosis, including higher drug use severity [28, 35, 36, 38]. In light of these findings, it would be interesting to conduct regular screenings for depressive symptoms to promptly identify changes in symptomatology scores when there is suspicion of an increase or intensification of drug use. By doing so, clinical attention could be improved through tailored interventions that address more explicitly depressive symptoms and prevent adverse outcomes in individuals with cocaine dependence. It is worth noting that this is particularly interesting in women, as literature suggests they are more likely than men to switch from abstinence to cocaine use [28]. Crits-Christoph and colleagues [20] found out that the presence of anhedonia symptoms was a strong predictor of poor treatment response; given these findings, it would be valuable to determine whether other specific depression symptoms have a relevant role in the treatment and prognosis of CUD, which would allow us to specify and tailor the treatment approach to very specific conditions.

Evidence on the predictive capacity of genetic markers in CUD is scant and more research is warranted to investigate the impact of genetic markers on both treatment and prognosis. Nonetheless, there is some evidence that suggests that the patients who carry the Val allele of the COMT Gene Val158met polymorphism are more likely to display three or more weeks of continuous abstinence, as well as a greater percentage of days of abstinence during treatment when undergo a CBT intervention [17].

Although there is no consensus regarding which treatment approach predicts better outcomes in CUD, the limited available data suggest that CM predicts long-term abstinence and higher rates of treatment retention. In other words, individuals who undergo CM have a better prognosis [11, 32, 33, 35].

There is a clear need to better elucidate the most important predictors of treatment outcomes in patients with CUD. In this regard, more research is warranted to study other factors, such as those related to emotion regulation. In real-life clinical practice, the important role of emotion regulation in patients undergoing cocaine detoxification treatment is readily apparent, yet we lack data in clinical settings on the role of emotion regulation in CUD [47]. In this regard, it would be interesting to conduct a study to determine whether this variable can predict treatment outcomes in CUD.

This study has some limitations. First, we screened three databases, and thus only manuscripts indexed in those databases were included, which means some relevant studies may have been missed. Subsequently, based on the conducted bibliographic searches, the authors are not aware of the existence of further studies on the current topic. However, given that these three databases are the largest and most important, the likelihood that we missed any important trials is low. Second, we limited our analysis to RCTs alone, excluding other article types, such as observational studies. The inclusion of other types of studies would have provided more data about the predictors evaluated in this review, or about other potential predictors of treatment outcomes that can be better analyzed through other study designs. Third, we included only manuscripts written in English or Spanish; by excluding studies written in other languages, we may have missed some relevant data. Fourth, due to the risk of bias assessment for the majority of the studies included in this review reporting some concerns, it is worth noting that these results cannot be easily generalized, therefore, they should be interpreted carefully.

A final limitation is that CM was a significant predictor of treatment outcomes but CBT was not. This finding was somewhat surprising, but it may be due to the study aims, which was to identify predictors of treatment outcomes rather than treatment efficiency. CM predicts treatment outcomes regardless of time point at which it is assessed, whereas CBT does not. Importantly, all of the main clinical practice guidelines suggest that CBT is a more efficient treatment for CUD in the long term, whereas CM is more efficient in the short term [2, 3, 5]. In fact, CM is considered the main treatment approach in addictions, especially at the beginning of the treatment. This is why CBT is not described as a predictive factor, even though it is commonly used to treat CUD.

## Conclusions

Younger age, more years of cocaine use, and higher craving levels were significant predictors of relapse and treatment dropout. By contrast, fewer withdrawal symptoms, greater baseline abstinence, and more self-efficacy were all predictive of longer duration of abstinence. The role of impulsivity as a predictor of CUD is unclear due to conflicting data, although the evidence generally suggests that higher impulsivity scores can predict more severe addiction and withdrawal symptoms, and earlier discontinuation of treatment.

### Supplementary Information

Additional file 1: Supplement 1. Completed PRISMA abstract checklist.

Additional file 2: Supplement 2. Completed PRISMA checklist for the narrative synthesis.

Additional file 3: Supplement 3. Search strategy, including the use of specific search terms, and reporting the number of identified reports for each search in the three consulted databases.

Additional file 4: Supplement 4. Reports that were excluded from the narrative synthesis due to not meeting the selection criteria. These excluded reports are categorized based on the specified criteria.

## Data Availability

All data analyzed or generated during this study are presented in the primary research articles reviewed or in this published article.

## Abbreviations

CUD: Cocaine Use Disorder
CM: Contingency Management
CBT: Cognitive Behavioral Therapy
CSSA: Cocaine Selective Severity Assessment
BIS-11: Barratt Impulsiveness Scale
RCT: Randomized Controlled Trial
PRISMA: Preferred Reporting Items for Systematic Reviews and Meta-Analyses
DSM-5: Diagnostic and Statistical Manual for Mental Disorders, Fifth Edition
MINI: Mini International Neuropsychiatric Interview
PICO: Population, Intervention, Comparison, Outcome
RoB-2: Cochrane Risk of Bias tool for randomized trials
LDA: Long Duration of Abstinence
ASI: Addiction Severity Index
BDI: Beck Depression Inventory
APD: Antisocial Personality Disorder
WCST: Wisconsin Card Sorting Test
fMRI: Functional Magnetic Resonance Imaging
dlPFC: Dorsolateral Prefrontal Cortex
COMT: Catechol-O-Methyltransferase
