# Supplementary material for: Predictors of cocaine use disorder treatment outcomes: a systematic review
Source: Syst Rev. 2024 May 8;13:124. doi: 10.1186/s13643-024-02550-z (PMC11077740; doi:10.1186/s13643-024-02550-z)
Supplement: Supplementary file 3 — Additional file 3: Supplement 3. Search strategy, including the use of specific search terms, and reporting the number of identified reports for each search in the three consulted databases. [file 13643_2024_2550_MOESM3_ESM.pdf]

PubMed

01/04/2023

#1	("cocaine"[MeSH Terms] OR "cocaine"[All Fields] OR "cocaine s"[All Fields] OR "cocaines"[All Fields] OR "cocainics"[All Fields]) AND (1000/1/1:2023/4/1[pdat])	46.073
#2	("cocaine"[MeSH Terms] OR "cocaine"[All Fields] OR "cocaine s"[All Fields] OR "cocaines"[All Fields] OR "cocainics"[All Fields]) AND ((clinicaltrial[Filter]) AND (1000/1/1:2023/4/1[pdat]))	1.952
#3	("cocaine"[MeSH Terms] OR "cocaine"[All Fields] OR "cocaine s"[All Fields] OR "cocaines"[All Fields] OR "cocainics"[All Fields]) AND ((clinicaltrial[Filter] OR randomizedcontrolledtrial[Filter]) AND (1000/1/1:2023/4/1[pdat]))	1.952
#4	(("cocaine"[MeSH Terms] OR "cocaine"[All Fields] OR "cocaine s"[All Fields] OR "cocaines"[All Fields] OR "cocainics"[All Fields]) AND ("treatment outcome"[MeSH Terms] OR ("treatment"[All Fields] AND "outcome"[All Fields]) OR "treatment outcome"[All Fields])) AND ((clinicaltrial[Filter] OR randomizedcontrolledtrial[Filter]) AND (1000/1/1:2023/4/1[pdat]))	731
#5	(("cocaine"[MeSH Terms] OR "cocaine"[All Fields] OR "cocaine s"[All Fields] OR "cocaines"[All Fields] OR "cocainics"[All Fields]) AND ("predictor"[All Fields] OR "predictors"[All Fields])) AND ((clinicaltrial[Filter] OR randomizedcontrolledtrial[Filter]) AND (1000/1/1:2023/4/1[pdat]))	101
#6	(("cocaine"[MeSH Terms] OR "cocaine"[All Fields] OR "cocaine s"[All Fields] OR "cocaines"[All Fields] OR "cocainics"[All Fields]) AND ("treatment outcome"[MeSH Terms] OR ("treatment"[All Fields] AND "outcome"[All Fields]) OR "treatment outcome"[All Fields]) AND ("predictor"[All Fields] OR "predictors"[All Fields])) AND ((clinicaltrial[Filter] OR randomizedcontrolledtrial[Filter]) AND (1000/1/1:2023/4/1[pdat]))	63

Web of Science

01/04/2023

#1	ALL=(cocaine)	63.511
#2	ALL=(cocaine) and Article (Document Types)	48.756
#3	(ALL=(cocaine)) AND ALL=(treatment outcome)	3.347
#4	(ALL=(cocaine)) AND ALL=(treatment outcome) and Article (Document Types)	2.910
#5	(ALL=(cocaine)) AND ALL=(predictors)	1.816
#6	(ALL=(cocaine)) AND ALL=(predictors) and Article (Document Types)	1.646
#7	((ALL=(cocaine)) AND ALL=(predictors)) AND ALL=(treatment outcome)	1.725
#8	(ALL=(cocaine)) AND ALL=(predictors)) AND ALL=(treatment outcome) and Article (Document Types)	490

APA PsychINFO (Proquest)

01/04/2023

#1	cocaine' AND rtype.exact("Journal Article") AND me.exact("Clinical Trial")<br>AND pd(<20210824)	356
#2	cocaine' AND (treatment outcome) AND rtype.exact("Journal Article") AND me.exact("Clinical Trial") AND pd(<20210824)	159
#3	cocaine' AND predictors AND rtype.exact("Journal Article") AND me.exact("Clinical Trial") AND pd(<20210824)	20
#4	cocaine' AND (treatment outcome) AND predictors AND rtype.exact("Journal Article") AND me.exact("Clinical Trial") AND pd(<20210824)	13
