# Supplementary material for: Predictors of cocaine use disorder treatment outcomes: a systematic review
Source: Syst Rev. 2024 May 8;13:124. doi: 10.1186/s13643-024-02550-z (PMC11077740; doi:10.1186/s13643-024-02550-z)
Supplement: Supplementary file 4 — Additional file 4: Supplement 4. Reports that were excluded from the narrative synthesis due to not meeting the selection criteria. These excluded reports are categorized based on the specified criteria. [file 13643_2024_2550_MOESM4_ESM.pdf]

Reasons for exclusion	Reference
Studies different from an RCT	Adelson M, Linzy S, Peles E. Characteristics and Outcome of Male and Female Methadone Maintenance Patients: MMT in Tel Aviv and Las Vegas. Subst Use & Misuse. 2018; https://doi.org/10.1080/10826084.2017.1298619
	Adelson M, Smith D, Peles E. Trend differences over 20 years between two methadone maintenance clinics, one with and one without cannabis legalization. J Addict Dis. 2020; https://doi.org/10.1080/10550887.2020.1848248
	Afriandi I, Kongsin S, Jiamton S, Intaraprasong B, Riono P. Factors associated with illicit injection drug use among methadone maintenance treatment patients in indonesia. Southeast Asian J Trop Med Public Health. 2020;51:26–35.
	Aharonovich E, Amrhein PC, Bisaga A, Nunes E V., Hasin DS. Cognition, Commitment Language, and Behavioral Change Among Cocaine-Dependent Patients. Psychol Addict Behav. 2008; https://doi.org/10.1037/a0012971
	Aharonovich E, Hasin D, Brooks A, Liu X, Bisaga A, Nunes E. Cognitive deficits predict low treatment retention in cocaine dependent patients. Drug Alcohol Depend. 2006; https://doi.org/10.1016/J.DRUGALCDEP.2005.08.003
	Alford DP, LaBelle CT, Kretsch N, Bergeron A, Winter M, Botticelli M, et al. Collaborative Care of Opioid-Addicted Patients in Primary Care Using Buprenorphine Five-Year Experience. Arch Intern Med. 2011;171:425–31.
	Alterman AI, Kampman K, Boardman CR, Cacciola JS, Rutherford MJ, McKay JR, et al. A cocaine-positive baseline urine predicts outpatient treatment attrition and failure to attain initial abstinence. Drug Alcohol Depend. 1997; https://doi.org/10.1016/S0376-8716(97)00049-5
	Alterman AI, Rutherford MJ, Cacciola JS, McKay JR, Boardman CR. Prediction of 7 months methadone

	maintenance treatment response by four measures of antisociality. Drug Alcohol Depend. 1998; https://doi.org/10.1016/S0376-8716(98)00015-5
	Amodeo M, Chassler D, Oettinger C, Labiosa W, Lundgren LM. Client retention in residential drug treatment for Latinos. Eval Program Plann. 2008; https://doi.org/10.1016/j.evalprogplan.2007.05.008
	Ashton H, Nodiyal A, Green D, Moore B, Heather N. Acupuncture or counselling: outcomes and predictors of treatment choice in a non-statutory addiction service. J Subst Use. 2009; https://doi.org/10.1080/14659890802659002
	Avants SK, Warburton LA, Margolin A. Spiritual and religious support in recovery from addiction among HIV-positive injection drug users. J Psychoactive Drugs. 2001; https://doi.org/10.1080/02791072.2001.10400467
	Bada HS, Bann CM, Bauer CR, Shankaran S, Lester B, LaGasse L, et al. Preadolescent behavior problems after prenatal cocaine exposure: Relationship between teacher and caretaker ratings (Maternal Lifestyle Study). Neurotoxicol Teratol. 2011; https://doi.org/10.1016/j.ntt.2010.06.005
	Bagra I, Krishnan V, Rao R, Agrawal A. Does Cannabis Use Influence Opioid Outcomes and Quality of Life Among Buprenorphine Maintained Patients? A Cross-sectional, Comparative Study. J Addict Med. 2018; https://doi.org/10.1097/ADM.0000000000000406
	Bargagli AM, Schifano P, Davoli M, Faggiano F, Perucci CA, Grp VeS. Determinants of methadone treatment assignment among heroin addicts on first admission to public treatment centres in Italy. Drug Alcohol Depend. 2005; https://doi.org/10.1016/j.drugalcdep.2005.01.014
	Basu D, Ghosh A, Sarkar S, Patra BN, Subodh BN, Mattoo SK. Initial treatment dropout in patients with substance use disorders attending a tertiary care de-addiction centre in north India. INDIAN J Med Res. 2017; https://doi.org/10.4103/ijmr.IJMR_1309_15

	Bauer LO. Predicting relapse to alcohol and drug abuse via quantitative electroencephalography. Neuropsychopharmacology. 2001; https://doi.org/10.1016/S0893-133X(01)00236-6
	Beadell NC, Thompson EM, Delashaw JB, Cetas JS. The deleterious effects of methamphetamine use on initial presentation and clinical outcomes in aneurysmal subarachnoid hemorrhage. J Neurosurg. 2012; https://doi.org/10.3171/2012.7.JNS12396
	Berg JM, Malte CA, Reger MA, Hawkins EJ. Medical Records Flag for Suicide Risk: Predictors and Subsequent Use of Care Among Veterans With Substance Use Disorders. Psychiatr Serv. 2018; https://doi.org/10.1176/appi.ps.201700545
	Bethea AR, Acosta MC, Haller DL. Patient versus therapist alliance: Whose perception matters? J Subst Abuse Treat. 2008; https://doi.org/10.1016/j.jsat.2007.09.007
	Bohnert KM, Ilgen MA, Louzon S, McCarthy JF, Katz IR. Substance use disorders and the risk of suicide mortality among men and women in the US Veterans Health Administration. Addiction. 2017; https://doi.org/10.1111/add.13774
	Bombardier CH, Hoekstra T, Dikmen S, Fann JR. Depression Trajectories during the First Year after Traumatic Brain Injury. J Neurotrauma. 2016; https://doi.org/10.1089/neu.2015.4349
	Boog M, Goudriaan AE, v d Wetering BJM, Polak M, Deuss H, Franken IHA. Rash Impulsiveness and Reward Sensitivity as predictors of treatment outcome in male substance dependent patients. Addict Behav. 2014; https://doi.org/10.1016/j.addbeh.2014.02.020
	Bottlender M, Soyka M. Outpatient alcoholism treatment: Predictors of outcome after 3 years. Drug Alcohol Depend. 2005; https://doi.org/10.1016/j.drugalcdep.2005.03.01
	Bowser BP, Lewis D, Dogan D. External Influences on Drug Treatment Interventions: East Palo Alto's Free-

	at-Last. J Addict Med. 2011; https://doi.org/10.1097/ADM.0b013e3181ea8d4a
	Brands B, Blake J, Marsh D. Impact of methadone program philosophy changes on early treatment outcomes. J Addict Dis. 2003; https://doi.org/10.1300/J069v22n03_03
	Brands B, Blake J, Marsh DC, Sproule B, Jeyapalan R, Li S. The impact of benzodiazepine use on methadone maintenance treatment outcomes. J Addict Dis. 2008; https://doi.org/10.1080/10550880802122620
	Brecht ML, von Mayrhauser C, Anglin MD. Predictors of relapse after treatment for methamphetamine use. J Psychoactive Drugs. 2000; https://doi.org/10.1080/02791072.2000.10400231
	Brecht ML, Herbeck D. Time to relapse following treatment for methamphetamine use: A long-term perspective on patterns and predictors. Drug Alcohol Depend. 2014; https://doi.org/10.1016/j.drugalcdep.2014.02.702
	Brecht ML, Huang D, Evans E, Hser Y-I. Polydrug use and implications for longitudinal research: Ten-year trajectories for heroin, cocaine, and methamphetamine users. Drug Alcohol Depend. 2008; https://doi.org/10.1016/j.drugalcdep.2008.01.021
	Britton PC, Conner KR. Suicide Attempts within 12 Months of Treatment for Substance Use Disorders. Suicide life-threatening Behav. 2010; https://doi.org/10.1521/suli.2010.40.1.14
	Brocato J, Wagner EF. Predictors of retention in an alternative-to-prison substance abuse treatment program. Crim Justice Behav. 2008; https://doi.org/10.1177/0093854807309429
	Broome KM, Joe GW, Simpson DD. HIV risk reduction in outpatient drug abuse treatment: Individual and geographic differences. AIDS Educ Prev. 1999;11:293–306.
	Brower KJ, Blow FC, Hill EM, Mudd SA. Treatment outcome of alcoholics with and without cocaine disorders. Alcohol Exp Res. 1994; https://doi.org/10.1111/j.1530-0277.1994.tb00939.x

	Butelman ER, Maremmani AGI, Bacciardi S, Chen CY, da Rosa JC, Kreek MJ. Non-medical Cannabis Self-Exposure as a Dimensional Predictor of Opioid Dependence Diagnosis: A Propensity Score Matched Analysis. Front psychiatry. 2018; https://doi.org/10.3389/fpsyt.2018.00283
	Cacciola JS, Rutherford MJ, Alterman AI, McKay JR, Snider EC. Personality disorders and treatment outcome in methadone maintenance patients. J Nerv Ment Dis. 1996; https://doi.org/10.1097/00005053-199604000-00006
	Callaghan RC, Cunningham JA. Intravenous and non-intravenous cocaine abusers admitted to inpatient detoxification treatment: a 3-year medical-chart review of patient characteristics and predictors of treatment re-admission. Drug Alcohol Depend. 2002; https://doi.org/10.1016/S0376-8716(02)00226-0
	Calvo F, Carbonell X, Valero R, Costa J, Turro O, Giralt C, et al. Early drop-outs and retentions in substance abuse outpatient clinics: a cross-sectional comparative study of factors that increase or decrease adherence. Aten Primaria. 2018; https://doi.org/10.1016/j.aprim.2017.06.006
	Carpenter KM, Martinez D, Vadhan NP, Barnes-Holmes D, Nunes E V. Measures of Attentional Bias and Relational Responding Are Associated with Behavioral Treatment Outcome for Cocaine Dependence. Am J Drug Alcohol Abuse. 2012; https://doi.org/ 10.3109/00952990.2011.643986
	Carroll KM, Power MED, Bryant K, Rounsaville BJ. One-year follow-up status of treatment-seeking cocaine abusers - psychopathology and dependence severity as predictors of outcome. J Nerv Ment Dis. 1993; https://doi.org/10.1097/00005053-199302000-00001
	Casares-Lopez MJ, Diaz-Mesa E, Garcia-Portilla P, Saiz P, Teresa Bobes-Bascaran M, Fonseca-Pedrero E, et al. Sixth version of the Addiction Severity Index: Assessing sensitivity to therapeutic change and retention predictors. Int J Clin Heal Psychol. 2011;11:495–508
	Jose Casares-Lopez M, Gonzalez-Menendez A, Festinger DS, Fernandez-Garcia P, Fernandez-Hermida JR,

	Secades R, et al. Predictors of retention in a drug-free unit/substance abuse treatment in prison. Int J Law Psychiatry. 2013; https://doi.org/10.1016/j.ijlp.2013.04.003
	Causey ST, Towe SL, Hartsock J, Xu Y, Meade CS. Perceived Healthcare Access among Persons with and without HIV Who Use Illicit Stimulants: The Role of Cumulative Risk. Subst Use & Misuse. 2021; https://doi.org/10.1080/10826084.2021.1928211
	Cavicchioli M, Prudenziati F, Movalli M, Ramella P, Maffei C. The Severity of Personality Pathology: A Risk Factor for Concurrent Substance Use Disorders in Alcohol Use Disorder. J Dual Diagn. 2019; https://doi.org/10.1080/15504263.2019.1612131
	Chang G, Meadows M-E, Jones JA, Antin JH, Orav EJ. Substance Use and Survival after Treatment for Chronic Myelogenous Leukemia (CML) or Myelodysplastic Syndrome (MDS). Am J Drug Alcohol Abuse. 2010; https://doi.org/10.3109/00952990903490758
	Chang G, Weiss AP, Orav EJ, Rauch SL. Predictors of frequent emergency department use among patients with psychiatric illness. Gen Hosp Psychiatry. 2014; https://doi.org/ 10.1016/j.genhosppsych.2014.09.010
	Charney DA, Zikos E, Gill KJ. Early recovery from alcohol dependence: Factors that promote or impede abstinence. J Subst Abuse Treat. 2010; https://doi.org/10.1016/j.jsat.2009.06.002
	Chasnoff IJ, Griffith DR, Freier C, Murray J. Cocaine polydrug use in pregnancy - 2-year follow-up. Pediatrics. 1992;89:284–9.
	Chun-Hung L, Tso-Jen W, Hsin-Pei T, Yu-Hsin L, Bell J. Familial expressed emotion among heroin addicts in methadone maintenance treatment: Does it matter? Addict Behav. 2015; https://doi.org/10.1016/j.addbeh.2015.01.014
	Church SH, Rothenberg JL, Sullivan MA, Bornstein G, Nunes E V. Concurrent substance use and outcome in combined behavioral and naltrexone therapy for opiate dependence. Am J Drug Alcohol Abuse. 2001;

	https://doi.org/10.1081/ADA-100104511
	Clark CB, McCullumsmith CB, Redmond N, Hardy S, Waesche MC, Osula G, et al. Factors associated with rapid failure in a Treatment Alternatives for Safer Communities (TASC) program. J Subst Abuse Treat. 2013; https://doi.org/10.1016/j.jsat.2013.05.007
	Collins C, Kohler C, DiClemente R, Wang MQ. Evaluation of the exposure effects of a theory-based street outreach HIV intervention on African-American drug users. Eval Program Plann. 1999; https://doi.org/10.1016/S0149-7189(99)00018-X
	Congia P, Orru MG, Masia M, Muscas E, Manca S. Predicting Outcome in Public Addiction Services Using Data Collected During Routinely Assessment Procedures. Int J Ment Health Addict. 2016; https://doi.org/10.1007/s11469-015-9587-5
	Coon GM, Pena D, Illich PA. Self-efficacy and substance abuse: Assessment using a brief phone interview. J Subst Abuse Treat. 1998; https://doi.org/10.1016/S0740-5472(97)00285-7
	Coviello DM, Cornish JW, Lynch KG, Boney TY, Clark CA, Lee JD, et al. A Multisite Pilot Study of Extended-Release Injectable Naltrexone Treatment for Previously Opioid-Dependent Parolees and Probationers. Subst Abus. 2012; https://doi.org/10.1080/08897077.2011.609438
	Cox GM, Comiskey CM. Does Concurrent Cocaine Use Compromise 1-Year Treatment Outcomes for Opiate Users?? Subst Use & Misuse. 2011; https://doi.org/10.3109/10826084.2010.501649
	Crist RC, Vickers-Smith R, Kember RL, Rentsch CT, Xu H, Edelman EJ, et al. Analysis of genetic and clinical factors associated with buprenorphine response. Drug Alcohol Depend. 2021; https://doi.org/10.1016/j.drugalcdep.2021.109013
	Crits-Christoph P, Markell HM, Gallop R, Gibbons MBC, McClure B, Rotrosen J. Predicting outcome of

	substance abuse treatment in a feedback study: Can recovery curves be improved upon? Psychother Res. 2015; https://doi.org/10.1080/10503307.2014.994146
	Cui Z, Hayashi K, Bach P, Dong H, Milloy MJ, Kerr T. Predictors of crystal methamphetamine use initiation or re-initiation among people receiving opioid agonist therapy: A prospective cohort study. Drug Alcohol Depend. 2022; https://doi.org/10.1016/j.drugalcdep.2022.109624
	Curran GM, Kirchner JE, Worley M, Rookey C, Booth BM. Depressive symptomatology and early attrition from intensive outpatient substance use treatment. J Behav Heal Serv & Res. 2002; https://doi.org/10.1007/BF02287700
	Czermainski FR, Lopes FM, Ornell F, Pinto Guimaraes LS, Von Diemen L, Kessler F, et al. Concurrent Use of Alcohol and Crack Cocaine is Associated with High Levels of Anger and Liability to Aggression. Subst Use & Misuse. 2020; https://doi.org/10.1080/10826084.2020.1756850
	da Cruz TA, da Cunha GN, de Moraes VP, Massarini R, Kawata Yoshida CM, Tenguam PT, et al. ICD-10 mental and behavioural disorders due to use of crack and powder cocaine as treated at a public psychiatric emergency service: An analysis of visit predictors. Int Rev Psychiatry. 2014; https://doi.org/10.3109/09540261.2014.928271
	Dacosta-Sánchez D, González-Ponce BM, Fernández-Calderón F, Sánchez-García M, Lozano OM. Retention in treatment and therapeutic adherence: How are these associated with therapeutic success? An analysis using real-world data. Int J Methods Psychiatr Res. 2022; https://doi.org/10.1002/mpr.1929
	Darker C, Sweeney B, El Hassan H, Kelly A, O' Connor S, Smyth B, et al. Non-attendance at counselling therapy in cocaine-using methadone-maintained patients: lessons learnt from an abandoned randomised controlled trial. Ir J Med Sci. 2012; https://doi.org/10.1007/s11845-011-0803-4
	Delic M. Inpatient management of ghb/gbl withdrawal. Psychiatr Danub. 2019;31:S354–6

	Dias AC, Araujo MR, Dunn J, Sesso RC, de Castro V, Laranjeira R. Mortality rate among crack/cocaine-dependent patients: A 12-year prospective cohort study conducted in Brazil. J Subst Abuse Treat. 2011; https://doi.org/10.1016/j.jsat.2011.03.008
	DiClemente CC, Corno CM, Graydon MM, Wiprovnick AE, Knoblach DJ. Motivational Interviewing, Enhancement, and Brief Interventions Over the Last Decade: A Review of Reviews of Efficacy and Effectiveness. Psychol Addict Behav. 2017; https://doi.org/10.1037/adb0000318
	Dong H, Hayashi K, Fairbairn N, Milloy M-J, DeBeck K, Wood E, et al. Long term pre-treatment opioid use trajectories in relation to opioid agonist therapy outcomes among people who use drugs in a Canadian setting. Addict Behav. 2021; https://doi.org/10.1016/j.addbeh.2020.106655
	Dowling N. Client characteristics associated with treatment attrition and outcome in female pathological gambling. Addict Res & Theory. 2009; https://doi.org/10.1080/16066350802346193
	Jiang D, David H, Min Z, Hser YI. Drug-abusing Offenders with Co-morbid Mental Disorders: Gender Differences in Problem Severity, Treatment Participation, and Recidivism. Biomed Environ Sci. 2013; https://doi.org/10.3967/0895-3988.2013.01.004
	Ducray K, Byrne P, Burke C, Smyth BP. A comparison of the drug use patterns, measures of needs and quality of life of methadone-maintained patients using and not using cocaine. HEROIN Addict Relat Clin Probl. 2011;13:27–37.
	Dunlap LJ, Zarkin GA, Lennox R, Bray JW. Do treatment services for drug users in outpatient drug-free treatment programs affect employment and crime? Subst Use & Misuse. 2007; https://doi.org/10.1080/10826080701409925
	Fitzpatrick RE, Rubenis AJ, Lubman DI, Verdejo-Garcia A. Cognitive deficits in methamphetamine addiction: Independent contributions of dependence and intelligence. Drug Alcohol Depend. 2020; https://doi.org/10.1016/j.drugalcdep.2020.107891

	Fletcher BW, Broome KM, Delany PJ, Shields J, Flynn PM. Patient and program factors in obtaining supportive services in DATOS. J Subst Abuse Treat. 2003; https://doi.org/10.1016/S0740-5472(03)00126-0
	Forster SE, Dickey MW, Forman SD. Regional cerebral blood flow predictors of relapse and resilience in substance use recovery: A coordinate-based meta-analysis of human neuroimaging studies. Drug Alcohol Depend. 2018; https://doi.org/10.1016/j.drugalcdep.2017.12.009
	Forster SE, Finn PR, Brown JW. Neural responses to negative outcomes predict success in community-based substance use treatment. Addiction. 2017; https://doi.org/10.1111/add.13734
	Franklin TR, Ehrman R, Lynch KG, Harper D, Sciortino N, O'Brien CP, et al. Menstrual cycle phase at quit date predicts smoking status in an NRT treatment trial: A retrospective analysis. J Womens Heal. 2008; https://doi.org/10.1089/jwh.2007.0423
	Franques P, Auriacombe M, Tignol J. Addiction and personality. Enceph Psychiatr Clin Biol Ther. 2000;26:68–78.
	Fridell M, Hesse M, Jaeger MM, Kuhlhorn E. Antisocial personality disorder as a predictor of criminal behaviour in a longitudinal study of a cohort of abusers of several classes of drugs: Relation to type of substance and type of crime. Addict Behav. 2008; https://doi.org/10.1016/j.addbeh.2008.01.001
	Froeliger B, Kozink R V, Rose JE, Behm FM, Salley AN, McClernon FJ. Hippocampal and striatal gray matter volume are associated with a smoking cessation treatment outcome: results of an exploratory voxel-based morphometric analysis. Psychopharmacology (Berl). 2010; https://doi.org/10.1007/s00213-010-1862-3
	Gadomski A, Riley M, Ramiza K, Onofrey L, Zinkievich R, Krupa N, et al. Treating Neonatal Abstinence Syndrome in a Rural Hospital: Lessons Learned. Acad Pediatr. 2018;18:425–9
	Gerra G, Borella F, Zaimovic A, Moi G, Bussandri M, Bubici C, et al. Buprenorphine versus methadone for opioid dependence: predictor variables for treatment outcome. Drug Alcohol Depend. 2004; https://doi.org/10.1016/j.drugalcdep.2003.11.017

	Ghitza UE, Preston KL, Epstein DH, Kuwabara H, Endres CJ, Bencherif B, et al. Brain Mu-Opioid Receptor Binding Predicts Treatment Outcome in Cocaine-Abusing Outpatients. Biol Psychiatry. 2010; https://doi.org/10.1016/j.biopsych.2010.05.003
	Godfrey CJ, Meyer TD, Boster KA, Vujanovic AA, Schmitz JM. Trauma and Emotion Regulation: Associations with Depressive Symptoms and Cocaine Use among Treatment-seeking Adults. Int J Ment Health Addict. 2022; https://doi.org/10.1007/s11469-021-00713-w
	Gomez-Bujedo J, Dominguez-Salas S, Juan Perez-Moreno P, Moraleda-Barreno E, Lozano OM. Reliability and validity evidence of a new interpretation bias task in patients diagnosed with drug use disorder: a preliminary study of the Word Association Task for Drug Use Disorder (WAT-DUD). Am J Drug Alcohol Abuse. 2019; https://doi.org/10.1080/00952990.2018.1559848
	Gonzalez-Alvarez S, Madoz-Gurpide A, Parro-Torres C, Hernandez-Huerta D, Ochoa Mangado E. Relationship between alcohol consumption, whether linked to other substance use or not, and antiretroviral treatment adherence in HIV plus patients. Adicciones. 2019; https://doi.org/10.20882/adicciones.916
	Gossop M, Marsden J, Stewart D, Kidd T. Changes in use of crack cocaine after drug misuse treatment: 4-5 year follow-up results from the National Treatment Outcome Research Study (NTORS). Drug Alcohol Depend. 2002; https://doi.org/10.1016/S0376-8716(01)00178-8
	Goudriaan AE, Oosterlaan J, De Beurs E, Van Den Brink W. The role of self-reported impulsivity and reward sensitivity versus neurocognitive measures of disinhibition and decision-making in the prediction of relapse in pathological gamblers. Psychol Med. 2008; https://doi.org/10.1017/S0033291707000694
	Gowin JL, Ernst M, Ball T, May AC, Sloan ME, Tapert SF, et al. Using neuroimaging to predict relapse in stimulant dependence: A comparison of linear and machine learning models. Neuroimage-Clinical. 2019; https://doi.org/10.1016/j.nicl.2019.101676

	Greenfield SF, Back SE, Lawson K, Brady KT. Substance Abuse in Women. Psychiatr Clin NORTH Am. 2010; https://doi.org/10.1016/j.psc.2010.01.004
	Grella CE, Hser YI, Hsieh SC. Predictors of drug treatment re-entry following relapse to cocaine use in DATOS. J Subst Abuse Treat. 2003; https://doi.org/10.1016/S0740-5472(03)00128-4
	Grella CE, Scott CK, Foss MA, Joshi V, Hser YI. Gender differences in drug treatment outcomes among participants in the Chicago Target Cities Study. Eval Program Plann. 2003; https://doi.org/10.1016/S0149-7189(03)00034-X
	Grella CE, Stein JA, Weisner C, Chi F, Moos R. Predictors of longitudinal substance use and mental health outcomes for patients in two integrated service delivery systems. Drug Alcohol Depend. 2010; https://doi.org/10.1016/j.drugalcdep.2010.02.013
	Grunbaum JA, Tortolero S, Weller N, Gingiss P. Cultural, social, and intrapersonal factors associated with substance use among alternative high school students. Addict Behav. 2000; https://doi.org/10.1016/S0306-4603(99)00006-4
	Guliyev C, İnce-Guliyev E, Ögel K. Predictors of Relapse to Alcohol and Substance Use: Are There Any Differences between 3 and 12 Months after Inpatient Treatment? J Psychoactive Drugs. 2022; https://doi.org/10.1080/02791072.2021.1976887
	Hambley J, Arbour S, Sivagnanasundaram L. Comparing outcomes for alcohol and drug abuse clients: A 6-month follow-up of clients who completed a residential treatment programme. J Subst Use. 2010; https://doi.org/10.3109/14659890903075066
	Harrell PT, Trenz RC, Scherer M, Martins SS, Latimer WW. A latent class approach to treatment readiness corresponds to a transtheoretical (''Stages of Change'') model. J Subst Abuse Treat. 2013; https://doi.org/10.1016/j.jsat.2013.04.004

	Harrison PA, Asche SE. Comparison of substance abuse treatment outcomes for inpatients and outpatients. J Subst Abuse Treat. 1999; https://doi.org/10.1016/S0740-5472(99)00004-5
	Hasin DS, Fink DS, Olfson M, Saxon AJ, Malte C, Keyes KM, et al. Substance use disorders and COVID-19: An analysis of nation-wide Veterans Health Administration electronic health records. Drug Alcohol Depend. 2022; https://doi.org/10.1016/j.drugalcdep.2022.109383
	Hayashi K, Wood E, Kerr T, Dong H, Nguyen P, Puskas CM, et al. Factors associated with optimal pharmacy refill adherence for antiretroviral medications and plasma HIV RNA non-detectability among HIV-positive crack cocaine users: a prospective cohort study. BMC Infect Dis. 2016; https://doi.org/10.1186/s12879-016-1749-y
	Haynes P. Drug using offenders in South London: Trends and outcomes. J Subst Abuse Treat. 1998; https://doi.org/10.1016/S0740-5472(97)00307-3
	Hechtman L. Predictors of long-term outcome in children with attention-deficit/hyperactivity disorder. Pediatr Clin North Am. 1999; https://doi.org/10.1016/S0031-3955(05)70171-1
	Heidebrecht F, MacLeod MB, Dawkins L. Predictors of heroin abstinence in opiate substitution therapy in heroin-only users and dual users of heroin and crack. Addict Behav. 2018; https://doi.org/10.1016/j.addbeh.2017.10.013
	Hohman MM, Shillington AM, Baxter HG. A comparison of pregnant women presenting for alcohol and other drug treatment by CPS status. CHILD Abus & Negl. 2003; https://doi.org/10.1016/S0145-2134(03)00008-5
	Howington JU, Kutz SC, Wilding GE, Awasthi D. Cocaine use as a predictor of outcome in aneurysmal subarachnoid hemorrhage. J Neurosurg. 2003; https://doi.org/10.3171/jns.2003.99.2.0271
	Hser YI, Joshi V, Anglin MD, Fletcher B. Predicting posttreatment cocaine abstinence for first-time

	admissions and treatment repeaters. Am J Public Health. 1999; https://doi.org/10.2105/AJPH.89.5.666
	Hser Y, Shen HK, Grella C, Anglin MD. Lifetime severity index for cocaine use disorder (LSI-cocaine) - A predictor of treatment outcomes. J Nerv Ment Dis. 1999; https://doi.org/10.1097/00005053-199912000-00006
	Hser YI, Evans E, Huang D, Brecht M-L, Li L. Comparing the dynamic course of heroin, cocaine, and methamphetamine use over 10 years. Addict Behav. 2008; https://doi.org/10.1016/j.addbeh.2008.07.024
	Hser Y-I, Kagihara J, Huang D, Evans E, Messina N. Mortality among substance-using mothers in California: a 10-year prospective study. Addiction. 2012; https://doi.org/10.1111/j.1360-0443.2011.03613.x
	Iannuzzi JC, Stapleton SM, Bababekov YJ, Chang D, Lancaster RT, Conrad MF, et al. Favorable impact of thoracic endovascular aortic repair on survival of patients with acute uncomplicated type B aortic dissection. J Vasc Surg. 2018; https://doi.org/10.1016/j.jvs.2018.04.034
	Ilgen MA, Harris AHS, Moos RH, Tiet QQ. Predictors of a suicide attempt one year after entry into substance use disorder treatment. Alcohol Exp Res. 2007; https://doi.org/10.1111/j.1530-0277.2007.00348.x
	Jackson AC, Dowling N, Thomas SA, Holt TA. Treatment careers in problem gambling: Factors associated with first treatment and treatment re-entry. Addict Res Theory. 2008; https://doi.org/10.1080/16066350802008793
	Jackson R, Wernicke R, Haaga DAF. Hope as a predictor of entering substance abuse treatment. Addict Behav. 2003; https://doi.org/10.1016/S0306-4603(01)00210-6
	Jarlais DCD, Hubbard R. Treatment for drug dependence. Proc Assoc Am Physicians. 1999; https://doi.org/10.1046/j.1525-1381.1999.09248.x
	Joe GW, Simpson DD, Broome KM. Retention and patient engagement models for different treatment modalities in DATOS. Drug Alcohol Depend. 1999; https://doi.org/10.1016/S0376-8716(99)00088-5

	Joe GW, Simpson DD, Dansereau DF, Rowan-Szal GA. Relationships between counseling rapport and drug abuse treatment outcomes. Psychiatr Serv. 2001; https://doi.org/10.1176/appi.ps.52.9.1223
	Joe GW, Simpson DD, Greener JM, Rowan-Szal GA. Development and validation of a client problem profile and index for drug treatment. Psychol Rep. 2004; https://doi.org/10.2466/pr0.95.1.215-234
	Joe GW, Flynn PM, Broome KM, Simpson DD. Patterns of drug use and expectations in methadone patients. Addict Behav. 2007; https://doi.org/10.1016/j.addbeh.2006.11.021
	Joe GW, Simpson DD, Rowan-Szal GA. Interaction of Counseling Rapport and Topics Discussed in Sessions with Methadone Treatment Clients. Subst Use & Misuse. 2009; https://doi.org/10.1080/10826080802525876
	Kabrhel C, Courtney DM, Camargo Jr. CA, Plewa MC, Nordenholz KE, Moore CL, et al. Factors Associated With Positive D-dimer Results in Patients Evaluated for Pulmonary Embolism. Acad Emerg Med. 2010; https://doi.org/10.1111/j.1553-2712.2010.00765.x
	Kader R, Seedat S, Koch JR, Parry CD. A preliminary investigation of the AUDIT and DUDIT in comparison to biomarkers for alcohol and drug use among HIV-infected clinic attendees in Cape Town, South Africa. Afr J Psychiatry. 2012; https://doi.org/10.4314/ajpsy.v15i5.43
	Kampman KM, Volpicelli JR, Mcginnis DE, Alterman AI, Weinrieb RM, D'Angelo L, et al. Reliability and validity of the Cocaine Selective Severity Assessment. Addict Behav. 1998; https://doi.org/10.1016/S0306-4603(98)00011-2
	Kasarabada ND, Anglin MD, Khalsa-Denison E, Paredes A. Differential effects of treatment modality on psychosocial functioning of cocaine-dependent men. J Clin Psychol. 1999; https://doi.org/10.1002/(SICI)1097-4679(199902)55:2<257::AID-JCLP13>3.3.CO;2-O
	Kellogg S, Melia D, Khuri E, Lin A, Ho A, Kreek MJ. Adolescent and young adult heroin patients: Drug use

	and success in methadone maintenance treatment. J Addict Dis. 2006; https://doi.org/10.1300/J069v25n03_03
	Kelly SM, O'Grady KE, Mitchell SG, Brown BS, Schwartz RP. Predictors of methadone treatment retention from a multi-site study: A survival analysis. Drug Alcohol Depend. 2011; https://doi.org/10.1016/j.drugalcdep.2011.01.008
	Kennedy AP, Epstein DH, Phillips KA, Preston KL. Sex differences in cocaine/heroin users: Drug-use triggers and craving in daily life. Drug Alcohol Depend. 2013; https://doi.org/10.1016/j.drugalcdep.2012.12.025
	Kenney SR, Anderson BJ, Bailey GL, Stein MD. Expectations about alcohol, cocaine, and benzodiazepine abstinence following inpatient heroin withdrawal management. Am J Addict. 2019; https://doi.org/10.1111/ajad.12834
	Kim TW, Palepu A, Cheng DM, Libman H, Saitz R, Samet JH. Factors associated with discontinuation of antiretroviral therapy in HIV-infected patients with alcohol problems. AIDS CARE-PSYCHOLOGICAL SOCIO-MEDICAL Asp AIDS/HIV. 2007; https://doi.org/10.1080/09540120701294245
	Kim TW, Samet JH, Cheng DM, Bernstein J, Wang N, German J, et al. The spectrum of unhealthy drug use and quality of care for hypertension and diabetes: a longitudinal cohort study. BMJ Open. 2015; https://doi.org/10.1136/bmjopen-2015-008508
	Kim TW, Samet JH, Cheng DM, Winter MR, Gelb Safran D, Saitz R. Primary care quality and addiction severity: A prospective cohort study. Health Serv Res. 2007; https://doi.org/10.1111/j.1475-6773.2006.00630.x
	King AC, Canada SA. Client-related predictors of early treatment drop-out in a substance abuse clinic exclusively employing individual therapy. J Subst Abuse Treat. 2004; https://doi.org/10.1016/S0740-5472(03)00210-1
	Kissin WB, Svikis DS, Moylan P, Haug NA, Stitzer ML. Identifying pregnant women at risk for early attrition

	from substance abuse treatment. J Subst Abuse Treat. 2004; https://doi.org/10.1016/j.jsat.2004.03.007
	Kleinman BP, Millery M, Scimeca M, Polissar NL. Predicting long-term treatment utilization among addicts entering detoxification: The contribution of help-seeking models. J Drug Issues. 2002; https://doi.org/10.1177/002204260203200109
	Knight DK, Logan SM, Simpson DD. Predictors of program completion for women in residential substance abuse treatment. Am J Drug Alcohol Abuse. 2001; https://doi.org/10.1081/ADA-100103116
	Knowlton AR, Latkin CA, Schroeder JR, Hoover DR, Ensminger M, Celentano DD. Longitudinal predictors of depressive symptoms among low income injection drug users. AIDS CARE-PSYCHOLOGICAL SOCIO-MEDICAL Asp AIDS/HIV. 2001; https://doi.org/10.1080/09540120120063197
	Kokkevi A. Psychosocial assessment in substance abuse and dependence. Curr Opin Psychiatry. 2001; https://doi.org/10.1097/00001504-200105000-00002
	Kumar V, Dolan RD, Yang AL, Jin DX, Banks PA, McNabb-Baltar J. Characteristics of 30-Day All-Cause Hospital Readmissions Among Patients with Acute Pancreatitis and Substance Use. Dig Dis Sci. 2022; https://doi.org/10.1007/s10620-022-07463-2
	Lambdin BH, Kral AH, Comfort M, Lopez AM, Lorvick J. Associations of criminal justice and substance use treatment involvement with HIV/HCV testing and the HIV treatment cascade among people who use drugs in Oakland, California. Addict Sci & Clin Pract. 2017; https://doi.org/10.1186/s13722-017-0078-9
	Landau J, Duncan Stanton M, Brinkman-Sull D, Ikle D, McCormick D, Garrett J, et al. Outcomes with the ARISE approach to engaging reluctant drug- and alcohol-dependent individuals in treatment. Am J Drug Alcohol Abuse. 2004; https://doi.org/10.1081/ADA-200037533
	Laudet AB, Stanick V. Predictors of motivation for abstinence at the end of outpatient substance abuse

	treatment. J Subst Abuse Treat. 2010; https://doi.org/10.1016/j.jsat.2010.01.007
	Laudet AB, Stanick V, Sands B. What could the program have done differently? A qualitative examination of reasons for leaving outpatient treatment. J Subst Abuse Treat. 2009; https://doi.org/10.1016/j.jsat.2009.01.001
	Ledgerwood DM, Lister JJ, LaLiberte B, Lundahl LH, Greenwald MK. Injection opioid use as a predictor of treatment outcomes among methadone-maintained opioid-dependent patients. Addict Behav. 2019; https://doi.org/10.1016/j.addbeh.2018.10.046
	Lemak CH, Alexander JA. Managed care and outpatient substance abuse treatment intensity. J Behav Heal Serv & Res. 2001; https://doi.org/10.1007/BF02287231
	Lipsitz SR, Fitzmaurice GM, Weiss RD. Using Multiple Imputation with GEE with Non-monotone Missing Longitudinal Binary Outcomes. Psychometrika. 2020; https://doi.org/10.1007/s11336-020-09729-y
	Lister JJ, Brown S, Greenwald MK, Ledgerwood DM. Gender-specific predictors of methadone treatment outcomes among African Americans at an urban clinic. Subst Abus. 2019; https://doi.org/10.1080/08897077.2018.1547810
	Lloyd MH, Akin BA. The disparate impact of alcohol, methamphetamine, and other drugs on family reunification. Child Youth Serv Rev. 2014; https://doi.org/10.1016/j.childyouth.2014.05.013
	Lopes-Rosa R, Kessler FP, Pianca TG, Guimaraes L, Ferronato P, Pagnussat E, et al. Predictors of early relapse among adolescent crack users. J Addict Dis. 2017; https://doi.org/10.1080/10550887.2017.1295670
	Lopez-Torrecillas F, Perales JC, Nieto-Ruiz A, Verdejo-Garcia A. Temperament and Impulsivity Predictors of Smoking Cessation Outcomes. PLoS One. 2014; https://doi.org/10.1371/journal.pone.0112440
	Lorenzoni V, Curzio O, Karakachoff M, Saponaro A, Sanza M, Mariani F, et al. The effects of the macro-environment on treatment retention for problem cocaine users. Int J Drug Policy. 2013;

	https://doi.org/10.1016/j.drugpo.2012.07.001
	Lowder EM, Zhou W, Peppard L, Bates R, Carr T. Supply-side predictors of fatal drug overdose in the Washington/Baltimore HIDTA region: 2016–2020. Int J Drug Policy. 2022; https://doi.org/10.1016/j.drugpo.2022.103902
	Lubman DI, Yucel M, Kettle JWL, Scaffidi A, MacKenzie T, Simmons JG, et al. Responsiveness to Drug Cues and Natural Rewards in Opiate Addiction Associations With Later Heroin Use. Arch Gen Psychiatry. 2009; https://doi.org/10.1001/archgenpsychiatry.2008.522
	Luchansky B, Krupski A, Stark K. Treatment response by primary drug of abuse: Does methamphetamine make a difference? J Subst Abuse Treat. 2007; https://doi.org/10.1016/j.jsat.2006.06.007
	Lundgren LM, Schilling RF, Ferguson F, Davis K, Amodeo M. Examining drug treatment program entry of injection drug users: human capital and institutional disaffiliation. Eval Program Plann. 2003; https://doi.org/10.1016/S0149-7189(03)00013-2
	Luo SX, Martinez D, Carpenter KM, Slifstein M, Nunes E V. Multimodal predictive modeling of individual treatment outcome in cocaine dependence with combined neuroimaging and behavioral predictors. Drug Alcohol Depend. 2014; https://doi.org/10.1016/J.DRUGALCDEP.2014.04.030
	Luo X, Zhang S, Hu S, Bednarski SR, Erdman E, Farr OM, et al. Error processing and gender-shared and -specific neural predictors of relapse in cocaine dependence. BRAIN. 2013; https://doi.org/10.1093/brain/awt040
	Mackay L, Bach P, Milloy M-J, Cui Z, Kerr T, Hayashi K. The relationship between crystal methamphetamine use and methadone retention in a prospective cohort of people who use drugs. Drug Alcohol Depend. 202; https://doi.org/10.1016/j.drugalcdep.2021.108844

	Mackin RS, Horner MD, Harvey RT, Stevens LA. The relationship between neuropsychological measures and employment problems in outpatients with substance abuse. Rehabil Psychol. 2005; https://doi.org/10.1037/0090-5550.50.2.158
	Magill M, Ray L, Kiluk B, Hoadley A, Bernstein M, Tonigan JS, et al. A Meta-Analysis of Cognitive-Behavioral Therapy for Alcohol or Other Drug Use Disorders: Treatment Efficacy by Contrast Condition. J Consult Clin Psychol. 2019; https://doi.org/10.1037/ccp0000447
	Magura S, Fong C, Staines GL, Cleland C, Foote J, Rosenblum A, et al. The combined effects of treatment intensity, self-help groups and patient attributes on drinking outcomes. J Psychoactive Drugs. 2005; https://doi.org/10.1080/02791072.2005.10399751
	Magura S, Rosenblum A, Fong C, Villano C, Richman B. Treating cocaine-using methadone patients: Predictors of outcomes in a psychosocial clinical trial. Subst Use Misuse. 2002; https://doi.org/10.1081/JA-120016225
	Majumder P, Sarkar S, Gupta R, Patra BN, Balhara YPS. Predictors of retention in treatment in a tertiary care de-addiction center. Indian J Psychiatry. 2016; https://doi.org/10.4103/0019-5545.174359
	Malta M, Magnanini MMF, Mello MB, Pascom ARP, Linhares Y, Bastos FI. HIV prevalence among female sex workers, drug users and men who have sex with men in Brazil: A Systematic Review and Meta-analysis. BMC Public Health. 2010; https://doi.org/10.1186/1471-2458-10-317
	Mandell W, Edelen MO, Wenzel SL, Dahl J, Ebener P. Do dimensions of therapeutic community treatment predict retention and outcomes? J Subst Abuse Treat. 2008; https://doi.org/10.1016/j.jsat.2007.10.004
	Manzoni P, Brochu S, Fischer B, Rehm J. Determinants of property crime among illicit opiate users outside of treatment across Canada. DEVIANT Behav. 2006; https://doi.org/10.1080/01639620600605705

	Marchi NC, Scherer JN, Pachado MP, Guimaraes LS, Siegmund G, de Castro MN, et al. Crack-cocaine users have less family cohesion than alcohol users. Rev Bras Psiquiatr. 2017; https://doi.org/10.1590/1516-4446-2016-2091
	Marco Mourino A, da Silva Moran A, Ortiz Seuma J, Sole Carbo C, Roget Alemany M, Sarriera Gracia C, et al. Predictors of adherence to treatment of chronic hepatitis C in drug-dependent Inmate Patients in four prisons in Barcelona, Spain. Rev Esp Salud Publica. 2010;84:423–31
	Marel C, Mills KL, Slade T, Darke S, Ross J, Teesson M. Modelling Long-Term Joint Trajectories of Heroin Use and Treatment Utilisation: Findings from the Australian Treatment Outcome Study. EClinicalMedicine. 2019; https://doi.org/10.1016/j.eclinm.2019.07.013
	Maremmani AGI, Aglietti M, Intaschi G, Bacciardi S. Substance Use/Dependence in Psychiatric Emergency Setting Leading to Hospitalization: Predictors of Continuity of Care. Int J Environ Res Public Health. 2022; https://doi.org/10.3390/ijerph19020760
	Martin RA, MacKinnon S, Johnson J, Rohsenow DJ. Purpose in life predicts treatment outcome among adult cocaine abusers in treatment. J Subst Abuse Treat. 2011; https://doi.org/ 10.1016/j.jsat.2010.10.002
	Martin TC, Josiah-Martin JA, Kosakoski J, Norton K, Sinnott T. A comparison of patients relapsing to addictive drug use with non-relapsing patients following residential addiction treatment in Antigua. West Indian Med J. 2005;54:196–201
	Martinez-Loredo V, Macipe V, Perez JME, Al-Halabi S. Clinical symptoms and personality traits predict subpopulations of treatment-seeking substance users. J Subst Abuse Treat. 2021; https://doi.org/10.1016/j.jsat.2021.108314
	McAweeney M, Rogers NL, Huddleston C, Moore D, Gentile JP. Symptom Prevalence of ADHD in a Community Residential Substance Abuse Treatment Program. J Atten Disord. 2010;

	https://doi.org/10.1177/1087054708329973
	McCarthy JE, Siney C, Shaw NJ, Ruben SM. Outcome predictors in pregnant opiate and polydrug users. Eur J Pediatr. 1999; https://doi.org/10.1007/s004310051193
	McHugh MJ, Gu H, Yang Y, Adinoff B, Stein EA. Executive control network connectivity strength protects against relapse to cocaine use. Addict Biol. 2017; https://doi.org/10.1111/adb.12448
	McKay JR, Foltz C, Leahy P, Stephens R, Orwin RG, Crowley EM. Step down continuing care in the treatment of substance abuse: correlates of participation and outcome effects. Eval Program Plann. 2004; https://doi.org/10.1016/j.evalprogplan.2004.04.005
	McKay JR, Gutman M, McLellan AT, Lynch KG, Ketterlinus R. Treatment services received in the CASAWORKS for families program. Eval Rev. 2003; https://doi.org/10.1177/0193841X03259028
	McLellan AT, Alterman AI, Metzger DS, Grissom GR, Woody GE, Luborsky L, et al. Similarity of outcome predictors across opiate, cocaine, and alcohol treatments - role of treatment services. J Consult Clin Psychol. 1994; https://doi.org/10.1037/0022-006X.62.6.1141
	McMahon RC. Personality, stress, and social support in cocaine relapse prediction. J Subst Abuse Treat. 2001; https://doi.org/10.1016/S0740-5472(01)00187-8
	McMahon RC, Enders C. Personality Disorder Factors Predict Recovery of Employment Functioning Among Treated Cocaine Abusers. Am J Drug Alcohol Abuse. 2009; https://doi.org/10.1080/00952990902825397
	Meghani SH, Wiedemer NL, Becker WC, Gracely EJ, Gallagher RM. Predictors of Resolution of Aberrant Drug Behavior in Chronic Pain Patients Treated in a Structured Opioid Risk Management Program. PAIN Med. 2009; https://doi.org/10.1111/j.1526-4637.2009.00643.x
	Miguel AQC, Kiluk BD, Roos CR, Babuscio TA, Nich C, Mari JJ, et al. Change in employment status and

	cocaine use treatment outcomes: A secondary analysis across six clinical trials. J Subst Abuse Treat. 2019; https://doi.org/10.1016/j.jsat.2019.09.002
	Miles DR, Svikis DS, Kulstad JL, Haug NA. Psychopathology in pregnant drug-dependent women with and without comorbid alcohol dependence. Alcohol Exp Res. 2001; https://doi.org/10.1097/00000374-200107000-00010
	Miller CL, Tyndall M, Spittal P, Li K, Palepu A, Schechter MT. Risk-taking behaviors among injecting drug users who obtain syringes from pharmacies, fixed sites, and mobile van needle exchanges. J URBAN Heal NEW YORK Acad Med. 2002; https://doi.org/10.1093/jurban/79.2.257
	Milligan CO, Nich C, Carroll KM. Ethnic differences in substance abuse treatment retention, compliance, and outcome from two clinical trials. Psychiatr Serv. 2004; https://doi.org/10.1176/appi.ps.55.2.167
	Mimiaga MJ, Reisner SL, Fontaine Y-M, Bland SE, Driscoll MA, Isenberg D, et al. Walking the line: Stimulant use during sex and HIV risk behavior among Black urban MSM. Drug Alcohol Depend. 2010; https://doi.org/10.1016/j.drugalcdep.2010.01.017
	Minkoff HL, Eisenberger-Matityahu D, Feldman J, Burk R, Clarke L. Prevalence and incidence of gynecologic disorders among women infected with human immunodeficiency virus. Am J Obstet Gynecol. 1999; https://doi.org/10.1016/S0002-9378(99)70653-8
	Moeeni M, Razaghi EM, Ponnet K, Torabi F, Shafiee SA, Pashaei T. Predictors of time to relapse in amphetamine-type substance users in the matrix treatment program in Iran: a Cox proportional hazard model application. BMC Psychiatry. 2016; https://doi.org/10.1186/s12888-016-0973-8
	Moeller SJ, Beebe-Wang N, Woicik PA, Konova AB, Maloney T, Goldstein RZ. Choice to view cocaine images predicts concurrent and prospective drug use in cocaine addiction. Drug Alcohol Depend. 2013; https://doi.org/10.1016/j.drugalcdep.2012.11.001

	Moos RH, Nichol AC, Moos BS. Risk factors for symptom exacerbation among treated patients with substance use disorders. Addiction. 2002; https://doi.org/10.1046/j.1360-0443.2002.00063.x
	Moraleda Barrenoa E, Dominguez-Salas S, Diaz-Batanero C, Lozano OM, Lorca Marin JA, Verdejo-Garcia A. Specific aspects of cognitive impulsivity are longitudinally associated with lower treatment retention and greater relapse in therapeutic community treatment. J Subst Abuse Treat. 2019; https://doi.org/10.1016/j.jsat.2018.10.004
	Morgenstern J, Bates ME. Effects of executive function impairment on change processes and substance use outcomes in 12-step treatment. J Stud Alcohol. 1999; https://doi.org/10.15288/jsa.1999.60.846
	Morrissey JP, Ellis AR, Gatz M, Amaro H, Reed BG, Savage A, et al. Outcomes for women with co-occurring disorders and trauma: Program and person-level effects. J Subst Abuse Treat. 2005; https://doi.org/10.1016/j.jsat.2004.08.012
	Morse SA, Watson C, MacMaster SA, Bride BE. Differences Between Older and Younger Adults in Residential Treatment for Co-Occurring Disorders. J Dual Diagn. 2015; https://doi.org/10.1080/15504263.2014.993263
	Moses TEH, Greenwald MK. History of regular nonmedical sedative and/or alcohol use differentiates substance-use patterns and consequences among chronic heroin users. Addict Behav. 2019; https://doi.org/10.1016/j.addbeh.2019.05.017
	Moses TE, Rhodes GL, Tavakoli E, Christensen CW, Amirsadri A, Greenwald MK. Predictors of Retention and Drug Use Among Patients With Opioid Use Disorder Transferred to a Specialty “Second Chance” Methadone Program. Subst Abus Res Treat. 2022; https://doi.org/10.1177/11782218221138335
	Mosli M, Parfitt J, Gregor J. Retrospective analysis of disease association and outcome in histologically confirmed ischemic colitis. J Dig Dis. 2013; https://doi.org/10.1111/1751-2980.12045

	Myers MG, Stewart DG, Brown SA. Progression from conduct disorder to antisocial personality disorder following treatment for adolescent substance abuse. Am J Psychiatry. 1998; https://doi.org/10.1176/ajp.155.4.479
	Neufeld K, King V, Peirce J, Kolodner K, Brooner R, Kidorf M. A comparison of 1-year substance abuse treatment outcomes in community syringe exchange participants versus other referrals. Drug Alcohol Depend. 2008; https://doi.org/10.1016/j.drugalcdep.2008.03.026
	Newton-Taylor B, Patra J, Gliksman L. Toronto drug treatment court: participant intake characteristics as predictors of ``successful'' program completion. J Drug Issues. 2009; https://doi.org/10.1177/002204260903900410
	Nwakeze PC, Magura S, Rosenblum A. Drug problem recognition, desire for help, and treatment readiness in a soup kitchen population. Subst Use & Misuse. 2002; https://doi.org/10.1081/JA-120002480
	Obialo CI, Bashir K, Goring S, Robinson B, Quarshie A, Al-Mahmoud A, et al. Dialysis ``No-Shows'' on saturdays: Implications of the weekly hemodialysis schedules on nonadherence and outcomes. J Natl Med Assoc. 2008; https://doi.org/10.1016/S0027-9684(15)31274-8
	Pagnin D, de Queiroz V, Saggese EG. Predictors of attrition from day treatment of adolescents with substance-related disorders. Addict Behav. 2005; https://doi.org/10.1016/j.addbeh.2004.09.013
	Palepu A, Tyndall MW, Leon H, Muller J, O'Shaughnessy M V, Schechter MT, et al. Hospital utilization and costs in a cohort of injection drug users. Can Med Assoc J. 2001;165:415–20
	Palmer RS, Murphy MK, Piselli A, Ball SA. Substance User Treatment Dropout from Client and Clinician Perspectives: A Pilot Study. Subst Use & Misuse. 2009; https://doi.org/10.1080/10826080802495237
	Panlilio V L, Stull SW, Kowalczyk WJ, Phillips KA, Schroeder JR, Bertz JW, et al. Stress, craving and mood

	as predictors of early dropout from opioid agonist therapy. Drug Alcohol Depend. 2019; https://doi.org/10.1016/j.drugalcdep.2019.05.026
	Paraherakis A, Charney DA, Palacios-Boix J, Gill K. An abstinence-oriented program for substance use disorders: Poorer outcome associated with opiate dependence. Can J PSYCHIATRY-REVUE Can Psychiatr. 2000; https://doi.org/10.1177/070674370004501009
	Passetti F, Clark L, Davis P, Mehta MA, White S, Checinski K, et al. Risky decision-making predicts short-term outcome of community but not residential treatment for opiate addiction. Implications for case management. Drug Alcohol Depend. 2011; https://doi.org/10.1016/j.drugalcdep.2011.02.015
	Patel RS, Manocha P, Patel J, Patel R, Tankersley WE. Cannabis Use Is an Independent Predictor for Acute Myocardial Infarction Related Hospitalization in Younger Population. J Adolesc Heal. 2020; https://doi.org/10.1016/j.jadohealth.2019.07.024
	Patkar AA, Gottheil E, Berrettini WH, Thornton CC, Hill KP, Weinstein SP. Relationship between platelet serotonin uptake sites and treatment outcome among African-American cocaine dependent individuals. J Addict Dis. 2003; https://doi.org/10.1300/J069v22n01_06
	Patkar AA, Murray HW, Mannelli P, Gottheil E, Weinstein SP, Vergare MJ. Pre-treatment measures of impulsivity, aggression and sensation seeking are associated with treatment outcome for African-American cocaine-dependent patients. J Addict Dis. 2004; https://doi.org/10.1300/J069v23n02_08
	Patkar A, Mannelli P, Certa K, Peindl K, Murray H, Vergare M, et al. Relationship of serum prolactin with severity of drug use and treatment outcome in cocaine dependence. Psychopharmacology (Berl). 2004; https://doi.org/10.1007/s00213-004-1856-0
	Patkar AA, Vergare MJ, Thornton CC, Weinstein SP, Murray HW, Leone FT. Nicotine dependence and treatment outcome among African American cocaine-dependent patients. Nicotine Tob Res. 2003;

	https://doi.org/10.1007/s00213-004-1856-0
	Patton T, Abramovitz D, Johnson D, Leas E, Nobles A, Caputi T, et al. Characterizing Help-Seeking Searches for Substance Use Treatment From Google Trends and Assessing Their Use for Infoveillance: Longitudinal Descriptive and Validation Statistical Analysis. J Med Internet Res. 2022; https://doi.org/10.2196/41527
	Pavia L, Di Blasi M, Cinquegrana A, Scioti E, Bussola T, Pasinelli A, et al. The Influence of Retention, Turnover, and Alliance on Process and Outcomes in Rolling Group Psychotherapy for Cocaine Disorder. Int J Group Psychother. 2016; https://doi.org/10.1080/00207284.2016.1176491
	Pavia L, Tosto C, Cinquegrana A, Scioti E, Bussola T, Cavani P. Rolling psychodynamic group for cocaine use disorder: A single-group study using multilevelmodels. J Groups Addict Recover. 2017; https://doi.org/10.1080/1556035X.2017.1313148
	Payne BE, Klein JW, Simon CB, James JR, Jackson SL, Merrill JO, et al. Effect of lowering initiation thresholds in a primary care-based buprenorphine treatment program. Drug Alcohol Depend. 2019; https://doi.org/10.1016/j.drugalcdep.2019.03.009
	Peles E, Schreiber S, Adelson M. Factors predicting retention in treatment: 10-year experience of a methadone maintenance treatment (MMT) clinic in Israel. Drug Alcohol Depend. 2006; https://doi.org/10.1016/j.drugalcdep.2005.09.004
	Peles E, Adelson M. Gender differences and pregnant women in a methadone maintenance treatment (MMT) clinic. J Addict Dis. 2006; https://doi.org/10.1300/J069v25n02_06
	Peles E, Linzy S, Kreek MJ, Adelson M. One-Year and Cumulative Retention as Predictors of Success in Methadone Maintenance Treatment: A Comparison of Two Clinics in the United States and Israel. J Addict Dis. 2008; https://doi.org/10.1080/10550880802324382

	Peles E, Schreiber S, Sason A, Adelson M. Similarities and changes between 15-and 24-year survival and retention rates of patients in a large medical-affiliated methadone maintenance treatment (MMT) center. Drug Alcohol Depend. 2018; https://doi.org/10.1016/j.drugalcdep.2017.11.034
	Peles E, Schreiber S, Sason A, Adelson M. Long Waiting Period to Enter Methadone Maintenance Treatment: Relation to Patient Characteristics and Outcome. Eur Addict Res. 2012; https://doi.org/10.1159/000336313
	Pelissier BMM, Camp SD, Gaes GG, Saylor WG, Rhodes W. Gender differences in outcomes from prison-based residential treatment. J Subst Abuse Treat. 2003; https://doi.org/10.1016/S0740-5472(02)00353-7
	Pelissier B, Camp SD, Motivans M. Staying in treatment: How much difference is there from prison to prison? Psychol Addict Behav. 2003; https://doi.org/10.1037/0893-164X.17.2.134
	Peretti-Watel P, Spire B, Lert F, Obadia Y, Grp V. Drug use patterns and adherence to treatment among HIV-positive patients: evidence from a large sample of French outpatients (ANRS-EN12-VESPA 2003). Drug Alcohol Depend. 2006; https://doi.org/10.1016/S0376-8716(06)80012-8
	Petry NM. A comparison of African American and non-Hispanic Caucasian cocaine-abusing outpatients. Drug Alcohol Depend. 2003; https://doi.org/10.1016/S0376-8716(02)00255-7
	Pettinati HM, Pierce JD, Belden PP, Meyers K. The relationship of axis II personality disorders to other known predictors of addiction treatment outcome. Am J Addict. 1999;8:136–47.
	Piz L, Maremrnani AGI, Rovai L, Bacciardi S, Rugani F, Maremmani I. Successful long-term (3-year) treatment of gambling with naltrexone. A case report. HEROIN Addict Relat Clin Probl. 2013;15:47–54.
	Poling J, Kosten TR, Sofuoglu M. Treatment outcome predictors for cocaine dependence. Am J Drug Alcohol Abuse. 2007; https://doi.org/10.1080/00952990701199416
	Proctor SL, Copeland AL, Kopak AM, Hoffmann NG, Herschman PL, Polukhina N. Outcome predictors for

	patients receiving methadone maintenance treatment: findings from a retrospective multi-site study. J Subst Use. 2016; https://doi.org/10.3109/14659891.2015.1118564
	Proctor SL, Copeland AL, Kopak AM, Hoffmann NG, Herschman PL, Polukhina N. Predictors of Patient Retention in Methadone Maintenance Treatment. Psychol Addict Behav. 2015; https://doi.org/10.1037/adb0000090
	Quinn B, Stoove M, Dietze P. One-year changes in methamphetamine use, dependence and remission in a community-recruited cohort. J Subst Use. 2016; https://doi.org/10.3109/14659891.2015.1018972
	Redko C, Rapp RC, Carlson RG. Pathways of substance users linking (or not) with treatment. J Drug Issues. 2007; https://doi.org/10.1177/002204260703700306
	Regier PS, Jagannathan K, Franklin TR, Wetherill RR, Langleben DD, Gawyrsiak M, et al. Sustained brain response to repeated drug cues is associated with poor drug-use outcomes. Addict Biol. https://doi.org/10.1111/adb.13028
	Reid SD, Simeon DT. Progression of dreams of crack cocaine abusers as a predictor of treatment outcome: A preliminary report. J Nerv Ment Dis. 2001; https://doi.org/10.1097/00005053-200112000-00007
	Ries RK, Yuodelis-Flores C, Comtois KA, Roy-Byrne PP, Russo JE. Substance-induced suicidal admissions to an acute psychiatric service: Characteristics and outcomes. J Subst Abuse Treat. 2008; https://doi.org/10.1016/j.jsat.2006.12.033
	Riley BJ. The role of homework in exposure-based CBT outcome for problem gambling. Int Gambl Stud. 2015; https://doi.org/10.1080/14459795.2015.1062532
	Rivera Mindt M, Arentoft A, Tureson K, Summers AC, Morris EP, Guzman V, et al. Disparities in Electronically Monitored Antiretroviral Adherence and Differential Adherence Predictors in Latinx and Non-

	Latinx White Persons Living with HIV. AIDS Patient Care STDS. 2020; https://doi.org/10.1089/apc.2019.0256
	Rodriguez N, Webb VJ. Multiple measures of juvenile drug court effectiveness: Results of a quasi-experimental design. Crime & Delinq. 2004; https://doi.org/10.1177/0011128703254991
	Rohsenow DJ, Martin RA, Eaton CA, Monti PM. Cocaine craving as a predictor of treatment attrition and outcomes after residential treatment for cocaine dependence. J Stud Alcohol Drugs. 2007; https://doi.org/10.15288/jsad.2007.68.641
	Roll JM, Higgins ST, Budney AJ, Bickel WK, Badger GJ. A comparison of cocaine-dependent cigarette smokers and non-smokers on demographic, drug use and other characteristics. Drug Alcohol Depend. 1996; https://doi.org/10.1016/0376-8716(96)01219-7
	Roll JM, Saules KK, Chudzynski JE, Sodano R. Relationship between tridimensional personality questionnaire scores and clinic attendance among cocaine abusing, buprenorphine maintained outpatients. Subst Use & Misuse. 2004; https://doi.org/10.1081/JA-120030898
	Rootman DB, Mann RE, Ferris LE, Chalin C, Adlaf E, Shuggi R. Predictors of completion status in a remedial program for male convicted drinking drivers. J Stud Alcohol. 2005; https://doi.org/10.15288/jsa.2005.66.423
	Ross S, Dermatis H, Levounis P, Galanter M. A comparison between dually diagnosed inpatients with and without axis II comorbidity and the relationship to treatment outcome. Am J Drug Alcohol Abuse. 2003; https://doi.org/10.1081/ADA-120020511
	Roux P, Carrieri PM, Cohen J, Ravaux I, Spire B, Gossop M, et al. Non-medical use of opioids among HIV-infected opioid dependent individuals on opioid maintenance treatment: the need for a more comprehensive approach. HARM Reduct J. 2011; https://doi.org/10.1186/1477-7517-8-31

	Rowell-Cunsolo TL, Sampong SA, Befus M, Mukherjee D V, Larson EL. Predictors of Illicit Drug Use Among Prisoners. Subst Use & Misuse. 2016; https://doi.org/10.3109/10826084.2015.1082594
	Roy E, Arruda N, Jutras-Aswad D, Berbiche D, Motta-Ochoa R, Bruneau J. Tranquilizer misuse among active cocaine users: Predictors of initiation. Drug Alcohol Rev. 2018; https://doi.org/10.1111/dar.12666
	Rubenis AJ, Fitzpatrick RE, Lubman I D, Verdejo-Garcia A. Sustained attention but not effort-based decision-making predicts treatment motivation change in people with methamphetamine dependence. J Subst Abuse Treat. 2018; https://doi.org/10.1016/j.jsat.2018.09.007
	Rubenis AJ, Fitzpatrick RE, Lubman DI, Verdejo-Garcia A. Working memory predicts methamphetamine hair concentration over the course of treatment: moderating effect of impulsivity and implications for dual-systems model. Addict Biol. 2019; https://doi.org/10.1111/adb.12575
	Rubin LH, Cook JA, Grey DD, Weber K, Wells C, Golub ET, et al. Perinatal Depressive Symptoms in HIV-Infected Versus HIV-Uninfected Women: A Prospective Study from Preconception to Postpartum. J WOMENS Heal. 2011; https://doi.org/10.1089/jwh.2010.2485
	Rudolph AE, Fernau DJ, Tobin KE, Latkin C. Individual and social network correlates of recent treatment for substance use disorders among persons who use drugs in Baltimore, MD (2014-2017). Drug Alcohol Depend. 2020; https://doi.org/10.1016/j.drugalcdep.2020.108278
	Ryoo H-J, Choo EK. Gender Differences in Emergency Department Visits and Detox Referrals for Illicit and Nonmedical Use of Opioids. West J Emerg Med. 2016; https://doi.org/10.5811/westjem.2016.2.29425
	Salahuddin M, Manzar MD, Pandi-Perumal SR, Bahammam AS. Emerging Challenges in COVID-19 With Substance Use Disorders. Addict Disord their Treat. 2021; https://doi.org/10.1097/ADT.0000000000000266
	Salamina G, Diecidue R, Vigna-Taglianti F, Jarre P, Schifano P, Bargagli AM, et al. Effectiveness of

	Therapies for Heroin Addiction in Retaining Patients in Treatment: Results From the VEdeTTE Study. Subst Use & Misuse. 2010; https://doi.org/10.3109/10826081003791932
	San L, Bernardo M, Gomez A, Pena M. Factors associated with relapse in patients with schizophrenia. Int J Psychiatry Clin Pract. 2013; https://doi.org/10.3109/13651501.2012.687452
	Sanchez-Hervas E, Secades-Villa R, Santonja Gomez FJ, Zacares Romaguera F, Garcia-Rodriguez O, Martin Yanez E, et al. Treatment dropout in cocaine addicts. Adicciones. 2010; https://doi.org/10.20882/adicciones.215
	Sanchez-Hervas E, Zacares Romaguera F, Santonja Gomez FJ, Secades-Villa R, Garcia-Rodriguez O, Martin Yanez E. Urine Testing During Treatment Predicts Cocaine Abstinence. J Psychoactive Drugs. 2010; https://doi.org/10.1080/02791072.2010.10400697
	Sanvisens A, Hernandez-Rubio A, Zuluaga P, Fuster D, Papaseit E, Galan S, et al. Long-Term Outcomes of Patients With Cocaine Use Disorder: A 18-years Addiction Cohort Study. Front Pharmacol. 2021; https://doi.org/10.3389/fphar.2021.625610
	Satyanarayana S, Safren SA, Rogers BG, Bainter SA, Christopoulos KA, Fredericksen RJ, et al. Estimating HIV transmissions in a large US clinic-based sample: effects of time and syndemic conditions. J Int AIDS Soc. 2021; https://doi.org/10.1002/jia2.25679
	Shankaran S, Bann C, Das A, Lester B, Bada H, Bauer CR, et al. Risk for obesity in adolescence starts in early childhood. J Perinatol. 2011; https://doi.org/10.1038/jp.2011.14
	Shor-Posner G, Lecusay R, Miguez-Burbano MJ, Quesada J, Rodriguez A, Ruiz P, et al. Quality of life measures in the Miami HIV-1 infected drug abusers cohort: Relationship to gender and disease status. J Subst Abuse. 2000; https://doi.org/10.1016/S0899-3289(00)00035-3

	Siegal HA, Li L, Rapp RC. Abstinence trajectories among treated crack cocaine users. Addict Behav. 2002; https://doi.org/10.1016/S0306-4603(01)00184-8
	Silva MR, Pereira JC, Costa RR, Dias JA, Guimaraes MDC, Leite ICG. Drug addiction and alcoholism as predictors for tuberculosis treatment default in Brazil: a prospective cohort study. Epidemiol Infect. 2017; https://doi.org/10.1017/S0950268817002631
	Smith JW, Frawley PJ. Treatment outcome of 600 chemically dependent patients treated in a multimodal inpatient program including aversion therapy and pentothal interviews. J Subst Abuse Treat. 1993; https://doi.org/10.1016/0740-5472(93)90021-S
	Sondhi A, Eastwood B. Assessing diversionary approaches for drug misusers in police custody in London: engagement and treatment outcomes as part of the Drug Intervention Programme. Addict Res & THEORY. 2021; https://doi.org/10.1080/16066359.2020.1784880
	Staines G, Magura S, Rosenblum A, Fong C, Kosanke N, Foote J, et al. Predictors of drinking outcomes among alcoholics. Am J Drug Alcohol Abuse. 2003; https://doi.org/10.1081/ADA-120018847
	Sugarman DE, Kaufman JS, Trucco EM, Brown JC, Greenfield SF. Predictors of drinking and functional outcomes for men and women following inpatient alcohol treatment. Am J Addict. 2014; https://doi.org/10.1111/j.1521-0391.2014.12098.x
	Sung HE, Belenko S, Feng L, Tabachnick C. Predicting treatment noncompliance clients: A theoretical and among criminal justice-mandated empirical exploration. J Subst Abuse Treat. 2004; https://doi.org/10.1016/S0740-5472(03)00144-2
	Tasic JK, Valkanou MK, Dukanovic B, Bankovic D, Janjic V. Relapse Risk Factors in Heroin Addicts Treated with Naltrexone and Naltrexone-Behavioural Psychotherapy. Int J Ment Health Addict. 2018; https://doi.org/10.1007/s11469-017-9782-7

	Teichner G, Horner MD, Harvey RT. Neuropsychological predictors of the attainment of treatment objectives in substance abuse patients. Int J Neurosci. 2001; https://doi.org/10.3109/00207450109149753
	Thomas VH, Melchert TP, Banken JA. Substance dependence and personality disorders: Comorbidity and treatment outcome in an inpatient treatment population. J Stud Alcohol. 1999; https://doi.org/10.15288/jsa.1999.60.271
	Tonigan JS, Beatty GK. Twelve-Step Program Attendance and Polysubstance Use: Interplay of Alcohol and Illicit Drug Use. J Stud Alcohol Drugs. 2011;72:864–71.
	Tonigan JS, Rice SL. Is It Beneficial to Have an Alcoholics Anonymous Sponsor? Psychol Addict Behav. 2010; https://doi.org/10.1037/a0019013
	Tracy SW, Kelly JE, Moos RH. The influence of partner status, relationship quality and relationship stability on outcomes following intensive substance-use disorder treatment. J Stud Alcohol. 2005; https://doi.org/10.15288/jsa.2005.66.497
	Tremeau F, Darreye A, Khidichian F, Weibel H, Kempf M, Greth P, et al. Efficacy of a French methadone maintenance treatment program. Enceph Psychiatr Clin Biol Ther. 2002;28:448–53.
	Tzilos GK, Rhodes GL, Ledgerwood DM, Greenwald MK. Predicting Cocaine Group Treatment Outcome in Cocaine-Abusing Methadone Patients. Exp Clin Psychopharmacol. 2009; https://doi.org/10.1037/A0016835
	Vayalapalli S, Fareed A, Byrd-Sellers J, Stout S, Casarella J, Drexler K. Predictors of Substance Abuse Treatment Outcome in Hospitalized Veterans. Am J Addict. 2013; https://doi.org/10.1111/j.1521-0391.2013.12050.x
	Verdejo-Garcia A, Betanzos-Espinosa P, Lozano OM, Vergara-Moragues E, Gonzalez-Saiz F, Fernandez-Calderon F, et al. Self-regulation and treatment retention in cocaine dependent individuals: A longitudinal

	study. Drug Alcohol Depend. 2012; https://doi.org/10.1016/j.drugalcdep.2011.09.025
	Verdejo-Garcia A, Garcia-Fernandez G, Dom G. Cognition and addiction. DIALOGUES Clin Neurosci. 2019; https://doi.org/10.31887/DCNS.2019.21.3/gdom
	Vergara-Moragues E, Gonzalez-Saiz F, Lozano-Rojas O, Fernandez Calderon F, Verdejo Garcia A, Betanzos Espinosa P, et al. Relationship between psychopathological comorbidity and outcomes variables in-treatment cocaine dependent subject in therapeutic community. Adicciones. 2013; https://doi.org/10.20882/adicciones.60
	Waesche MC, Clark CB, Cropsey KL. The Connection Between Thwarted Belongingness, Alcohol Consumption, Suicidal, and Homicidal Ideation in a Criminal Justice Sample. J Addict Med. 2016; https://doi.org/10.1097/ADM.0000000000000257
	Waisberg JL, Porter JE. Purpose in life and outcome of treatment for alcohol dependence. Br J Clin Psychol. 1994; https://doi.org/10.1111/J.2044-8260.1994.TB01093.X
	Walker R. Retention in Treatment-Indicator or Illusion: An Essay. Subst Use & Misuse. 2009; https://doi.org/10.1080/10826080802525967
	Walton MA, Cunningham R, Chermack ST, Tripathi S, Weber J, Maio RF, et al. Predictors of violence following Emergency Department visit for cocaine-related chest pain. Drug Alcohol Depend. 2009; https://doi.org/10.1016/j.drugalcdep.2008.07.001
	Wan L, Baldridge RM, Colby AM, Stanford MS. Association of P3 amplitude to treatment completion in substance dependent individuals. PSYCHIATRY Res. 2010; https://doi.org/10.1016/j.psychres.2009.01.033
	Wang Y, Zuo J, Hao W, Shen H, Zhang X, Deng Q, et al. Quality of Life in Patients With Methamphetamine Use Disorder: Relationship to Impulsivity and Drug Use Characteristics. Front PSYCHIATRY. 2020; https://doi.org/10.3389/fpsyt.2020.579302

	Wanigasooriya A, Connor JP, Young RMD, Feeney GFX, Gullo MJ. Development and validation of the Stimulant Refusal Self-Efficacy Questionnaire (SRSEQ) in stimulant users in treatment. Drug Alcohol Depend. 2021; https://doi.org/ 10.1016/j.drugalcdep.2021.109069
	Ware OD, Manuel JI, Huhn AS. Adults With Opioid and Methamphetamine Co-use Have Lower Odds of Completing Short-Term Residential Treatment Than Other Opioid Co-use Groups: A Retrospective Health Services Study. Front Psychiatry. 2021; https://doi.org/10.3389/fpsyt.2021.784229
	Warner LA, Alegria M, Canino G. Remission from drug dependence symptoms and drug use cessation among women drug users in Puerto Rico. Arch Gen Psychiatry. 2004; https://doi.org/10.1001/archpsyc.61.10.1034
	Wasserman DA, Weinstein MG, Havassy BE, Hall SM. Factors associated with lapses to heroin use during methadone maintenance. Drug Alcohol Depend. 1998; https://doi.org/10.1016/S0376-8716(98)00092-1
	Weinstein ZM, Kim HW, Cheng DM, Quinn E, Hui D, Labelle CT, et al. Long-term retention in Office Based Opioid Treatment with buprenorphine. J Subst Abuse Treat. 2017; https://doi.org/10.1016/j.jsat.2016.12.010
	Weiss R, Griffin M, Hufford C. Craving in hospitalized cocaine abusers as a predictor of outcome. Am J Drug Alcohol Abuse. 1995; https://doi.org/10.3109/00952999509002698
	Wemm SE, Larkin C, Hermes G, Tennen H, Sinha R. A day-by-day prospective analysis of stress, craving and risk of next day alcohol intake during alcohol use disorder treatment. Drug Alcohol Depend. 2019; https://doi.org/10.1016/j.drugalcdep.2019.107569
	Westhuis DJ, Gwaltney L, Hayashi R. Outpatient cocaine abuse treatment: Predictors of success. J Drug Educ. 2001; https://doi.org/10.2190/DQ7G-NBXR-6YQ7-1RXJ
	White WL, Campbell MD, Spencerc RD, Hoffman HA, Crissman B, DuPont RL. Patterns of Abstinence or Continued Drug Use Among Methadone Maintenance Patients and Their Relation to Treatment Retention. J

	Psychoactive Drugs. 2014; https://doi.org/10.1080/02791072.2014.901587
	Williams N, Bossert N, Chen Y, Jaanimagi U, Markatou M, Talal AH. Influence of social determinants of health and substance use characteristics on persons who use drugs pursuit of care for hepatitis C virus infection. J Subst Abuse Treat. 2019; https://doi.org/10.1016/j.jsat.2019.04.009
	Williamson A, Darke S, Ross J, Teesson M. The effect of persistence of cocaine use on 12-month outcomes for the treatment of heroin dependence. Drug Alcohol Depend. 2006; https://doi.org/10.1016/j.drugalcdep.2005.08.010
	Williamson A, Darke S, Ross J, Teesson M. The effect of baseline cocaine use on treatment outcomes for heroin dependence over 24 months: Findings from the Australian Treatment Outcome Study. J Subst Abuse Treat. 2007; https://doi.org/10.1016/j.jsat.2006.12.009
	Wu LJ, Altshuler SJ, Short RA, Roll JM. Predicting drug court outcome among amphetamine-using participants. J Subst Abuse Treat. 2012; https://doi.org/10.1016/j.jsat.2011.09.008
	Young SY, Delevoye-Turrell Y, van Hoof JJJ, Goudriaan AE, Seedat S. Association between motor timing and treatment outcomes in patients with alcohol and/or cocaine use disorder in a rehabilitation program. BMC Psychiatry. 2016;16.
	Young SY, Kidd M, van Hoof JJM, Seedat S. Prognostic Value of Motor Timing in Treatment Outcome in Patients With Alcohol- and/or Cocaine Use Disorder in a Rehabilitation Program. Front Psychol. 2018; https://doi.org/10.3389/fpsyg.2018.01945
	Zanis DA, Mulvaney F, Coviello D, Alterman AI, Savitz B, Thompson W. The effectiveness of early parole to substance abuse treatment facilities on 24-month criminal recidivism. J Drug Issues. 2003; https://doi.org/10.1177/002204260303300109

	Zemore SE, Kaskutas LA. Development and validation of the Alcoholics Anonymous Intention Measure (AAIM). Drug Alcohol Depend. 2009; https://doi.org/10.1016/j.drugalcdep.2009.04.019
	Zorick T, Mandelkern MA, Brody AL. A Naturalistic Study of the Association Between Antidepressant Treatment and Outcome of Smoking Cessation Treatment. J Clin Psychiatry. 2014; https://doi.org/10.4088/JCP.14m09012
Subjects aged < age 18	Battjes RJ, Gordon MS, O'Grady KE, Kinlock TW, Carswell MA. Factors that predict adolescent motivation for substance abuse treatment. J Subst Abuse Treat. 2003; https://doi.org/10.1016/S0740-5472(03)00022-9
	Chakrabarti A, Woody GE, Griffin ML, Subramaniam G, Weiss RD. Predictors of buprenorphine-naloxone dosing in a 12-week treatment trial for opioid-dependent youth: Secondary analyses from a NIDA Clinical Trials Network study. Drug Alcohol Depend. 2010; https://doi.org/10.1016/J.DRUGALCDEP.2009.10.014
	Gray JC, Padovano HT, Wemm SE, Miranda Jr. R. Predictors of Topiramate Tolerability in Heavy Cannabis-Using Adolescents and Young Adults: A Secondary Analysis of a Randomized, Double-Blind, Placebo-Controlled Trial. J Clin Psychopharmacol. 2018; https://doi.org/10.1097/JCP.0000000000000843
	McCamant LE, Zani BG, McFarland BH, Gabriel RM. Prospective validation of substance abuse severity measures from administrative data. Drug Alcohol Depend. 2007; https://doi.org/10.1016/j.drugalcdep.2006.04.016
	Strantz IH, Welch SP. Postpartum women in outpatient drug abuse treatment: Correlates of retention/completion. J Psychoactive Drugs. 1995; https://doi.org/10.1080/02791072.1995.10471701
Subjects without cocaine use disorder	Abramsohn Y, Peles E, Potik D, Schreiber S, Adelson M. Sense of Coherence as a Stable Predictor for Methadone Maintenance Treatment (MMT) Outcome. J Psychoactive Drugs. 2009; https://doi.org/10.1080/02791072.2009.10400535

	Acosta MC, Marsch LA, Xie H, Guarino H, Aponte-Melendez Y. A Web-Based Behavior Therapy Program Influences the Association Between Cognitive Functioning and Retention and Abstinence in Clients Receiving Methadone Maintenance Treatment. J Dual Diagn. 2012; https://doi.org/10.1080/15504263.2012.723317
	Adinoff B, Carmody TJ, Walker R, Donovan DM, Brigham GS, Winhusen TM. Decision-making processes as predictors of relapse and subsequent use in stimulant-dependent patients. Am J Drug Alcohol Abuse. 2016; https://doi.org/10.3109/00952990.2015.1106550
	Aguiar P, Neto D, Lambaz R, Chick J, Ferrinho P. Prognostic Factors During Outpatient Treatment for Alcohol Dependence: Cohort Study with 6 months of Treatment Follow-up. ALCOHOL Alcohol. 2012; https://doi.org/10.1093/alcalc/ags097
	Ahmadi J, Kampman KM, Oslin DM, Pettinati HM, Dackis C, Sparkman T. Predictors of treatment outcome in outpatient cocaine and alcohol dependence treatment. Am J Addict. 2009; https://doi.org/10.1080/10550490802545174
	Angelo FN, McDonell MG, Lewin MR, Srebnik D, Lowe J, Roll J, et al. Predictors of stimulant abuse treatment outcomes in severely mentally ill outpatients. Drug Alcohol Depend. 2013; https://doi.org/10.1016/j.drugalcdep.2012.11.017
	Apelt SM, Scherbaum N, Soyka M. Induction and switch to buprenorphine-naloxone in opioid dependence treatment: Predictive value of the first four weeks. HEROIN Addict Relat Clin Probl. 2014;16:87–97.
	Assadi SM, Radgoodarzi R, Ahmadi-abhari SA. Baclofen for maintenance treatment of opioid dependence : A. 2003;10.
	Avants SK, Margolin A, McKee S. A path analysis of cognitive, affective, and behavioral predictors of treatment response in a methadone maintenance program. J Subst Abuse. 2000; https://doi.org/10.1016/S0899-3289(00)00022-5

	Bauer LO, Covault J, Harel O, Das S, Gelernter J, Anton R, et al. Variation in GABRA2 predicts drinking behavior in project MATCH subjects. Alcohol Exp Res. 2007; https://doi.org/10.1111/j.1530-0277.2007.00517.x
	Brensilver M, Heinzerling KG, Swanson A-N, Shoptaw SJ. Placebo-Group Responders in Methamphetamine Pharmacotherapy Trials: The Role of Immediate Establishment of Abstinence. Exp Clin Psychopharmacol. 2012; https://doi.org/10.1037/a0029210
	Bunting AM, Krawczyk N, Choo TH, Pavlicova M, McNeely J, Tofighi B, et al. Polysubstance use before and during treatment with medication for opioid use disorder: Prevalence and association with treatment outcomes. J Subst Abuse Treat. 2022; https://doi.org/10.1016/j.jsat.2022.108830
	Carroll KM, Ball SA, Martino S, Nich C, Babuscio TA, Nuro KF, et al. Computer-assisted delivery of cognitive-behavioral therapy for addiction: A randomized trial of CBT4CBT. Am J Psychiatry. 2008; https://doi.org/10.1176/appi.ajp.2008.07111835
	Cornelius JR, Salloum IM, Lynch K, Clark DB, John Manna J. Treating the substance-abusing suicidal patient. Ann N Y Acad Sci. 2001; https://doi.org/10.1111/J.1749-6632.2001.TB05799.X
	Dreifuss JA, Griffin ML, Frost K, Fitzmaurice GM, Potter JS, Fiellin DA, et al. Patient characteristics associated with buprenorphine/naloxone treatment outcome for prescription opioid dependence: Results from a multisite study. Drug Alcohol Depend. 2013; https://doi.org/10.1016/j.drugalcdep.2012.12.010
	Downey KK, Helmus TC, Schuster CR. Treatment of heroin-dependent poly-drug abusers with contingency management and buprenorphine maintenance. Exp Clin Psychopharmacol. 2000; https://doi.org/10.1037//1064-1297.8.2.176
	Dunn KE, Harrison JA, Leoutsakos J-M, Han D, Strain EC. Continuous Abstinence During Early Alcohol Treatment is Significantly Associated with Positive Treatment Outcomes, Independent of Duration of

	Abstinence. ALCOHOL Alcohol. 2017; https://doi.org/10.1093/alcalc/agw059
	Erickson JR, Stevens S, McKnight P, Figueredo AJ. Willingness for treatment as a predictor of retention and outcomes. J Addict Dis. 1996;14:135–50.
	Evans EA, Zhu Y, Yoo C, Huang D, Hser YI. Criminal justice outcomes over 5 years after randomization to buprenorphine-naloxone or methadone treatment for opioid use disorder. Addiction. 2019; https://doi.org/10.1111/ADD.14620
	Feng N, Lin C, Hsieh J, Rou K, Li L. Family Related Factors and Concurrent Heroin Use in Methadone Maintenance Treatment in China. Subst Use & Misuse. 2018; https://doi.org/10.1080/10826084.2018.1424913
	Fink BC, Steele VR, Maurer MJ, Fede SJ, Calhoun VD, Kiehl KA. Brain potentials predict substance abuse treatment completion in a prison sample. BRAIN Behav. 2016; https://doi.org/10.1002/brb3.501
	Ford JD, Hawke J, Alessi S, Ledgerwood D, Petry N. Psychological trauma and PTSD symptoms as predictors of substance dependence treatment outcomes. Behav Res Ther. 2007;45:2417–31.
	Ghitza UE, Epstein DH, Preston KL. Nonreporting of cannabis use: Predictors and relationship to treatment outcome in methadone maintained patients. Addict Behav. 2007;32:938–49.
	Glasner-Edwards S, Marinelli-Casey P, Hillhouse M, Ang A, Mooney LJ, Rawson R, et al. Depression Among Methamphetamine Users Association With Outcomes From the Methamphetamine Treatment Project at 3-Year Follow-Up. J Nerv Ment Dis. 2009; https://doi.org/10.1097/NMD.0b013e31819db6fe
	Gonzalez G, Desai R, Sofuoglu M, Poling J, Oliveto A, Gonsai K, et al. Clinical efficacy of gabapentin versus tiagabine for reducing cocaine use among cocaine dependent methadone-treated patients. Drug Alcohol Depend. 2007; https://doi.org/10.1016/j.drugalcdep.2006.07.003
	Gordon MS, Kinlock TW, Schwartz RP, O'Grady KE. A randomized clinical trial of methadone maintenance

	for prisoners: findings at 6 months post-release. Addiction. 2008; https://doi.org/10.1111/j.1360-0443.2008.002238.x
	Grella CE, Anglin MD, Wugalter SE. Patterns and predictors of cocaine and crack use by clients in standard and enhanced methadone maintenance treatment. Am J Drug Alcohol Abuse. 1997; https://doi.org/10.3109/00952999709001685
	Gryczynski J, Kinlock TW, Kelly SM, O'Grady KE, Gordon MS, Schwartz RP. Opioid Agonist Maintenance for Probationers: Patient-Level Predictors of Treatment Retention, Drug Use, and Crime. Subst Abus. 2012; https://doi.org/10.1080/08897077.2011.616816
	Gryczynski J, Schwartz R, O'Grady K, Jaffe J. Treatment entry among individuals on a waiting list for methadone maintenance. Am J Drug Alcohol Abuse. 2009; https://doi.org/10.1080/00952990902968577
	Gryczynski J, Schwartz R, O'Grady K, Jaffe J. Treatment entry among individuals on a waiting list for methadone maintenance. Am J Drug Alcohol Abuse. 2009; https://doi.org/10.3109/00952990903322865
	Hall SM, Shi Y, Humfleet GL, Munoz RF, Reus VI, Prochaska JJ. Smoking cessation abstinence goal in treatment-seeking smokers. Addict Behav. 2015; https://doi.org/10.1016/J.addbeh.2014.11.012
	Hartzler B, Witkiewitz K, Villarroel N, Donovan D. Self-Efficacy Change as a Mediator of Associations Between Therapeutic Bond and One-Year Outcomes in Treatments for Alcohol Dependence. Psychol Addict Behav. 2011; https://doi.org/10.1037/a0022869
	Hatch-Maillette M, Wells EA, Doyle SR, Brigham GS, Daley D, DiCenzo J, et al. Predictors of 12-Step Attendance and Participation for Individuals With Stimulant Use Disorders. J Subst Abuse Treat. 2016; https://doi.org/10.1016/j.jsat.2016.06.007
	Hawkins EJ, Baer JS, Kivlahan DR. Concurrent monitoring of psychological distress and satisfaction measures

	as predictors of addiction treatment retention. J Subst Abuse Treat. 2008; https://doi.org/10.1016/j.jsat.2007.10.001
	Heinz AJ, Wu J, Witkiewitz K, Epstein DH, Preston KL. Marriage and relationship closeness as predictors of cocaine and heroin use. Addict Behav. 2009; https://doi.org/10.1016/j.addbeh.2008.10.020
	Helmus TC, Downey KK, Arfken CL, Henderson MJ, Schuster CR. Novelty seeking as a predictor of treatment retention for heroin dependent cocaine users. Drug Alcohol Depend. 2001; https://doi.org/10.1016/S0376-8716(00)00153-8
	Herrmann ES, Cooper ZD, Bedi G, Ramesh D, Reed SC, Comer SD, et al. Varenicline and nabilone in tobacco and cannabis co-users: effects on tobacco abstinence, withdrawal and a laboratory model of cannabis relapse. Addict Biol. 2019; https://doi.org/10.1111/adb.12664
	Hser YI, Huang D, Teruya C, Anglin MD. Diversity of drug abuse treatment utilization patterns and outcomes. Eval Program Plann. 2004; https://doi.org/10.1016/j.evalprogplan.2003.07.002
	Kampman KM, Pettinati HM, Volpicelli JR, Oslin DM, Lipkin C, Sparkman T, et al. Cocaine Dependence Severity Predicts Outcome in Outpatient Detoxification from Cocaine and Alcohol. Am J Addict. 2004; https://doi.org/10.1080/10550490490265389
	Kedia S, Williams C. Predictors of substance abuse treatment outcomes in Tennessee. J Drug Educ. 2003; https://doi.org/10.2190/RD7B-MDED-MEPJ-G7CD
	Kelly SM, O'Grady KE, Jaffe JH, Gandhi D, Schwartz RP. Improvements in Outcomes in Methadone Patients on Probation/Parole Regardless of Counseling Early in Treatment. J Addict Med. 2013; https://doi.org/10.1097/ADM.0b013e318284a0c1
	Killeen TK, Wen CC, Neelon B, Baker N. Predictors of Treatment Completion among Women Receiving

	Integrated Treatment for Comorbid Posttraumatic Stress and Substance Use Disorders. Subst Use Misuse . 2023; https://doi.org/10.1080/10826084.2023.2170183
	Kim SJ, Marsch LA, Guarino H, Acosta MC, Aponte-Melendez Y. Predictors of outcome from computer-based treatment for substance use disorders: Results from a randomized clinical trial. Drug Alcohol Depend. 2015;157:174–8.
	Kinlock TW, Gordon MS, Schwartz RP, O'Grady KE. A study of methadone maintenance for male prisoners. Crim Justice Behav. 2008; https://doi.org/10.1177/0093854807309111
	Kosten T, Sofuoglu M, Poling J, Gonsai K, Oliveto A. Desipramine treatment for cocaine dependence in buprenorphine- or methadone-treated patients: Baseline urine results as predictor of response. Am J Addict. 2005; https://doi.org/10.1080/10550490590899817
	Landabaso Vazquez M, Iraurgi Castillo I, Manuel Jimenez-Lerma J, Hormaechea Beldarrain JA, Gutierrez-Fraile M. Clinical Trial on the Use of Olanzapine in Reducing the Consumption of Cocaine in Methadone Maintenance Programmes. HEROIN Addict Relat Clin Probl. 2009;11:21–9.
	Levine AR, Lundahl LH, Ledgerwood DM, Lisieski M, Rhodes GL, Greenwald MK. Gender-Specific Predictors of Retention and Opioid Abstinence During Methadone Maintenance Treatment. J Subst Abuse Treat. 2015; https://doi.org/10.1016/j.jsat.2015.01.009
	Lions C, Carrieri MP, Michel L, Mora M, Marcellin F, Morel A, et al. Predictors of non-prescribed opioid use after one year of methadone treatment: An attributable-risk approach (ANRS-Methaville trial). Drug Alcohol Depend. 2014; https://doi.org/10.1016/J.DRUGALCDEP.2013.10.018
	Lister JJ, Greenwald MK, Ledgerwood DM. Baseline risk factors for drug use among African-American patients during first-month induction/stabilization on methadone. J Subst Abuse Treat. 2017; https://doi.org/10.1016/j.jsat.2017.04.007

	Lopez-Nunez C, Secades-Villa R, Pena-Suarez E, Fernandez-Artamendi S, Weidberg S. Income Levels and Response to Contingency Management for Smoking Cessation. Subst Use & Misuse. 2017; https://doi.org/10.1080/10826084.2016.1264973
	Mancino MJ, McGaugh J, Feldman Z, Poling J, Oliveto A. Effect of PTSD Diagnosis and Contingency Management Procedures on Cocaine Use in Dually Cocaine- and Opioid-Dependent Individuals Maintained on LAAM: A Retrospective Analysis. Am J Addict. 2010; https://doi.org/10.1111/j.1521-0391.2009.00025.x
	Marques A, Formigoni M. Comparison of individual and group cognitive-behavioral therapy for alcohol and/or drug-dependent patients. Addiction. 2001; https://doi.org/10.1046/j.1360-0443.2001.9668355.x
	Marsch LA, Bickel WK, Badger GJ, Jacobs EA. Buprenorphine treatment for opioid dependence: The relative efficacy of daily, twice and thrice weekly dosing. Drug Alcohol Depend. 2005; https://doi.org/10.1016/J.DRUGALCDEP.2004.08.011
	Marsch LA, Stephens MAC, Mudric T, Strain EC, Bigelow GE, Johnson RE. Predictors of outcome in LAAM, buprenorphine, and methadone treatment for opioid dependence. Exp Clin Psychopharmacol. 2005;https://doi.org/10.1037/1064-1297.13.4.29
	McCollum EE, Nelson TS, Lewis RA, Trepper TS. Partner relationship quality and drug use as predictors of women's substance abuse treatment outcome. Am J Drug Alcohol Abuse. 2005; https://doi.org/10.1081/ADA-200047906
	McHugh RK, Murray HW, Hearon BA, Pratt EM, Pollack MH, Safren SA, et al. Predictors of Dropout from Psychosocial Treatment in Opioid-Dependent Outpatients. Am J Addict. 2013; https://doi.org/10.1111/j.1521-0391.2013.00317.x
	McKay JR, Foltz C, Stephens RC, Leahy PJ, Crowley EM, Kissin W. Predictors of alcohol and crack cocaine use outcomes over a 3-year follow-up in treatment seekers. J Subst Abuse Treat. 2005;

	https://doi.org/10.1016/j.jsat.2004.10.010
	McPherson S, Packer RR, Cameron JM, Howell DN, Roll JM. Biochemical Marker of Use Is a Better Predictor of Outcomes Than Self-Report Metrics in a Contingency Management Smoking Cessation Analog Study. Am J Addict. 2014; https://doi.org/10.1111/j.1521-0391.2013.12059.x
	Millery M, Kleinman BP, Polissar NL, Millman RB, Scimeca M. Detoxification as a gateway to long-term treatment: assessing two interventions. J Subst Abuse Treat. 2002; https://doi.org/10.1016/S0740-5472(02)00246-5
	Mojarrad M, Samet JH, Cheng DM, Winter MR, Saitz R. Marijuana use and achievement of abstinence from alcohol and other drugs among people with substance dependence: A prospective cohort study. Drug Alcohol Depend. 2014; https://doi.org/10.1016/j.drugalcdep.2014.06.006
	Moore BA, Budney AJ. Tobacco smoking in marijuana-dependent outpatients. J Subst Abuse. 2001; https://doi.org/10.1016/S0899-3289(01)00093-1
	Moore BA, Budney AJ. Abstinence at intake for marijuana dependence treatment predicts response. Drug Alcohol Depend. 2002; https://doi.org/10.1016/S0376-8716(02)00079-0
	Morgenstern J, Bux D, Labouvie E, Blanchard KA, Morgan TJ. Examining mechanisms of action in 12-step treatment: The role of 12-step cognitions. J Stud Alcohol. 2002; https://doi.org/10.15288/jsa.2002.63.665
	Nielsen DA, Hamon SC, Kosten TR. The κ-opioid receptor gene as a predictor of response in a cocaine vaccine clinical trial. Psychiatr Genet. 2013; https://doi.org/10.1097/YPG.0000000000000008
	Nwakeze PC, Magura S, Rosenblum A, Joseph H. Service outcomes of peer consumer advocacy for soup kitchen guests. J Soc Serv Res. 2000; https://doi.org/10.1300/J079v27n02_02
	Oviedo-Joekes E, Sordo L, Guh D, Marsh DC, Lock K, Brissette S, et al. Predictors of non-use of illicit heroin

	in opioid injection maintenance treatment of long-term heroin dependence. Addict Behav. 2015; https://doi.org/10.1016/J.ADDBEH.2014.10.003
	Palis H, Guh D, MacDonald S, Harrison S, Brissette S, Marsh DC, et al. Longitudinal patterns of cocaine use among patients receiving injectable hydromorphone or diacetylmorphine for the treatment of opioid use disorder: A growth curve modeling approach. Drug Alcohol Depend. 2021; https://doi.org/10.1016/j.drugalcdep.2020.108333
	Patkar AA, Thornton CC, Mannelli P, Hill KP, Gottheil E, Vergare MJ, et al. Comparison of pretreatment characteristics and treatment outcomes for alcohol-, cocaine-, and multisubstance-dependent patients. J Addict Dis. 2004; https://doi.org/10.1300/J069v23n01_08
	Peirce JM, Petry NM, Stitzer ML, Blaine J, Kellogg S, Satterfield F, et al. Effects of lower-cost incentives on stimulant abstinence in methadone maintenance treatment - A national drug abuse treatment clinical trials network study. Arch Gen Psychiatry. 2006; https://doi.org/10.1001/archpsyc.63.2.201
	Peirce JM, Petry NM, Roll JM, Kolodner K, Krasnansky J, Stabile PQ, et al. Correlates of Stimulant Treatment Outcome Across Treatment Modalities. Am J Drug Alcohol Abuse. 2009; https://doi.org/10.1080/00952990802455444
	Penzenstadler L, Kolly S, Rothen S, Khazaal Y, Kramer U. Effects of substance use disorder on treatment process and outcome in a ten-session psychiatric treatment for borderline personality disorder. Subst Abus Treat Prev POLICY. 2018; https://doi.org/10.1186/s13011-018-0145-6
	Peters EN, Petry NM, LaPaglia DM, Reynolds B, Carroll KM. Delay Discounting in Adults Receiving Treatment for Marijuana Dependence. Exp Clin Psychopharmacol. 2013; https://doi.org/10.1037/a0030943
	Petry NM, Alessi SM, Hanson T, Sierra S. Randomized trial of contingent prizes versus vouchers in cocaine-using methadone patients. J Consult Clin Psychol. 2007; https://doi.org/10.1037/0022-006X.75.6.983

	Petry NM, Roll JM. Amount of Earnings During Prize Contingency Management Treatment Is Associated With Posttreatment Abstinence Outcomes. Exp Clin Psychopharmacol. 2011; https://doi.org/10.1037/a0024261
	Pirard S, Sharon E, Kang SK, Angarita GA, Gastfriend DR. Prevalence of physical and sexual abuse among substance abuse patients and impact on treatment outcomes. Drug Alcohol Depend. 2005; https://doi.org/10.1016/j.drugalcdep.2004.09.005
	Preston KL, Silverman K, Higgins ST, Brooner RK, Montoya I, Schuster CR, et al. Cocaine use early in treatment predicts outcome in a behavioral treatment program. J Consult Clin Psychol. 1998; https://doi.org/10.1037/0022-006X.66.4.691
	Ram A, Tuten M, Chisolm MS. Cigarette Smoking Reduction in Pregnant Women With Opioid Use Disorder. J Addict Med. 2016; https://doi.org/10.1097/ADM.0000000000000186
	Rawson RA, Gonzales R, Greenwell L, Chalk M. Process-of-Care Measures as Predictors of Client Outcome Among a Methamphetamine-Dependent Sample at 12-and 36-Month Follow-ups. J Psychoactive Drugs. 2012; https://doi.org/10.1080/02791072.2012.718653
	Ray GT, Weisner CM, Mertens JR. Relationship between use of psychiatric services and five-year alcohol and drug treatment outcomes. Psychiatr Serv. 2005; https://doi.org/10.1176/appi.ps.56.2.164
	Reiber C, Ramirez A, Parent D, Rawson RA. Predicting treatment success at multiple timepoints in diverse patient populations of cocaine-dependent individuals. Drug Alcohol Depend. 2002; https://doi.org/10.1016/S0376-8716(02)00103-5
	Ridenour TA, Kirisci L, Tarter RE, Vanyukov MM. Could a continuous measure of individual transmissible risk be useful in clinical assessment of substance use disorder? Findings from the National Epidemiological Survey on Alcohol and Related Conditions. Drug Alcohol Depend. 2011;

	https://doi.org/10.1016/j.drugalcdep.2011.05.018
	Rohsenow DJ, Miranda R, McGeary JE, Monti PM. Family History and Antisocial Traits Moderate Naltrexone's Effects on Heavy Drinking in Alcoholics. Exp Clin Psychopharmacol. 2007; https://doi.org/10.1037/1064-1297.15.3.272
	Roos CR, Kiluk BD, McHugh RK, Carroll KM. Evaluating a longitudinal mediation model of perceived stress, depressive symptoms, and substance use treatment outcomes. Psychol Addict Behav. 2020; https://doi.org/10.1037/ADB0000581
	Roux P, Lions C, Michel L, Cohen J, Mora M, Marcellin F, et al. Predictors of Non-adherence to Methadone Maintenance Treatment in Opioid-dependent Individuals: Implications for Clinicians. Curr Pharm Des. 2014; https://doi.org/10.2174/13816128113199990623
	Roux P, Lions C, Vilotitch A, Michel, Mora M, Maradan G, et al. Correlates of cocaine use during methadone treatment: Implications for screening and clinical management (ANRS Methaville study). Harm Reduct J. 2016;13.
	Rowan-Szal GA, Bartholomew NG, Chatham LR, Simpson DD. A combined cognitive behavioral intervention for cocaine-using methadone clients. J Psychoactive Drugs. 2005; https://doi.org/10.1080/02791072.2005.10399750
	Sanchez-Mazas P. Therapeutic evolution of drug addiction patients. Med Hyg (Geneve). 2003;61:1434–8.
	Saxon AJ, Wells EA, Fleming C, Jackson TR, Calsyn DA. Pre-treatment characteristics, program philosophy and level of ancillary services as predictors of methadone maintenance treatment outcome. Addiction. 1996; https://doi.org/10.1046/J.1360-0443.1996.918119711.X
	Sayre SL, Schmitz JM, Stotts AL, Averill PM, Rhoades HM, Grabowski JJ. Determining predictors of attrition

	in an outpatient substance abuse program. Am J Drug Alcohol Abuse. 2002; https://doi.org/10.1081/ADA-120001281
	Schaub MP, Haug S, Wenger A, Berg O, Sullivan R, Beck T, et al. Can reduce - the effects of chat-counseling and web-based self-help, web-based self-help alone and a waiting list control program on cannabis use in problematic cannabis users: a randomized controlled trial. BMC Psychiatry. 2013; https://doi.org/10.1186/1471-244X-13-305
	Secades-Villa R, Pericot-Valverde I, Weidberg S. Relative reinforcing efficacy of cigarettes as a predictor of smoking abstinence among treatment-seeking smokers. Psychopharmacology (Berl). 2016; https://doi.org/10.1007/s00213-016-4350-6
	Shearer J, Darke S, Rodgers C, Slade T, van Beek I, Lewis J, et al. A double-blind, placebo-controlled trial of modafinil (200 mg/day) for methamphetamine dependence. Addiction. 2009; https://doi.org/10.1111/j.1360-0443.2008.02437.x
	Stahler GJ, Shipley TF, Bartelt D, DuCette JP, Shandler IW. Evaluating alternative treatments for homeless substance-abusing men: Outcomes and predictors of success. J Addict Dis. 1995; https://doi.org/10.1300/J069v14n04_09
	Stanton CA, Kumar PN, Moadel AB, Cunningham CO, Schechter CB, Kim RS, et al. A Multicenter Randomized Controlled Trial of Intensive Group Therapy for Tobacco Treatment in HIV-Infected Cigarette Smokers. J Acquir Immune Defic Syndr. 2020; https://doi.org/10.1097/QAI.0000000000002271
	Stitzer ML, Petry N, Peirce J, Kirby K, Killeen T, Roll J, et al. Effectiveness of abstinence-based Incentives: Interaction with intake stimulant test results. J Consult Clin Psychol. 2007; https://doi.org/10.1037/0022-006X.75.5.805
	Sugarman DE, Nich C, Carroll KM. Coping Strategy Use Following Computerized Cognitive-Behavioral

	Therapy for Substance Use Disorders. Psychol Addict Behav. 2010; https://doi.org/10.1037/a0021584
	Sullivan LE, Moore BA, O'Connor PG, Barry DT, Chawarski MC, Schottenfeld RS, et al. The Association between Cocaine Use and Treatment Outcomes in Patients Receiving Office-Based Buprenorphine/Naloxone for the Treatment of Opioid Dependence. Am J Addict. 2010; https://doi.org/10.1111/j.1521-0391.2009.00003.x
	Tate SR, Mrnak-Meyer J, Shriver CL, Atkinson JH, Robinson SK, Brown SA. Predictors of Treatment Retention for Substance-Dependent Adults with Co-occurring Depression. Am J Addict. 2011; https://doi.org/10.1111/j.1521-0391.2011.00137.x
	Thomas Jr. PS, Nielsen EM, Spellicy CJ, Harding MJ, Ye A, Patriquin M, et al. The OPRD1 rs678849 variant influences outcome of disulfiram treatment for cocaine dependency in methadone-maintained patients. Psychiatr Genet. 2021; https://doi.org/10.1097/YPG.0000000000000279
	Tuten M, Shadur JM, Stitzer M, Jones HE. A Comparison of Reinforcement Based Treatment (RBT) versus RBT plus Recovery Housing (RBTRH). J Subst Abuse Treat. 2017; https://doi.org/10.1016/J.JSAT.2016.09.001
	Tuten M, Fitzsimons H, Hochheimer M, Jones HE, Chisolm MS. The Impact of Early Substance Use Disorder Treatment Response on Treatment Outcomes Among Pregnant Women With Primary Opioid Use. J Addict Med. 2018; https://doi.org/10.1097/ADM.0000000000000397
	Van Horn DHA, Rennert L, Lynch KG, McKay JR. Social Network Correlates of Participation in Telephone Continuing Care for Alcohol Dependence. Am J Addict. 2014; https://doi.org/10.1111/j.1521-0391.2014.12128.x

	Vujanovic AA, Smith LJ, Green C, Lane SD, Schmitz JM. Mindfulness as a predictor of cognitive-behavioral therapy outcomes in inner-city adults with posttraumatic stress and substance dependence. Addict Behav. 2020; https://doi.org/10.1016/j.addbeh.2019.106283
	Webster JM, Staton-Tindall M, Dickson MF, Wilson JF, Leukefeld CG. Twelve-month employment intervention outcomes for drug-involved offenders. Am J Drug Alcohol Abuse. 2014; https://doi.org/10.3109/00952990.2013.858722
	Weisner C, Ray GT, Mertens JR, Satre DD, Moore C. Short-term alcohol and drug treatment outcomes predict long-term outcome. Drug Alcohol Depend. 2003; https://doi.org/10.1016/S0376-8716(03)00167-4
	Wendt DC, Hallgren KA, Daley DC, Donovan DM. Predictors and outcomes of twelve-step sponsorship of stimulant users: Secondary analyses of a multisite randomized clinical trial. J Stud Alcohol Drugs. 2017; https://doi.org/10.15288/JSAD.2017.78.287
	Wilens TE, Adler LA, Tanaka Y, Xiao F, D'Souza DN, Gutkin SW, et al. Correlates of alcohol use in adults with ADHD and comorbid alcohol use disorders: exploratory analysis of a placebo-controlled trial of atomoxetine. Curr Med Res Opin. 2011; https://doi.org/10.1185/03007995.2011.628648
	Witbrodt J, Kaskutas LA. Does diagnosis matter? Differential effects of 12-step participation and social networks on abstinence. Am J Drug Alcohol Abuse. 2005; https://doi.org/10.1081/ADA-68486
	Witkiewitz K, Marlatt GA. Modeling the complexity of post-treatment drinking: It's a rocky road to relapse. Clin Psychol Rev. 2007; https://doi.org/10.1016/j.cpr.2007.01.002
	Yip SW, Scheinost D, Potenza MN, Carroll KM. Connectome-Based Prediction of Cocaine Abstinence. Am J Psychiatry. 2019; https://doi.org/10.1176/appi.ajp.2018.17101147
	Ziedonis DM, Kosten TR. Depression as a prognostic factor for pharmacological treatment of cocaine

	dependence. Psychopharmacol Bull. 1991;27:337–43.
	Zhu Y, Evans EA, Mooney LJ, Saxon AJ, Kelleghan A, Yoo C, et al. Correlates of Long-Term Opioid Abstinence After Randomization to Methadone Versus Buprenorphine/Naloxone in a Multi-Site Trial. J NEUROIMMUNE Pharmacol. 2018; https://doi.org/10.1007/s11481-018-9801-x
Subjects not in outpatient treatment	Alterman AI, McKay JR, Mulvaney FD, Cnaan A, Cacciola JS, Tourian KA, et al. Baseline prediction of 7-month cocaine abstinence for cocaine dependence patients. Drug Alcohol Depend. 2000; https://doi.org/10.1016/S0376-8716(99)00124-6
	Bamaby L, Gibson RC. Factors Affecting Completion of a 28-day inpatient Substance Abuse Treatment Programme at the University Hospital of the West Indies. WEST INDIAN Med J. 2008;57:364–8.
	Bashiri M, Mancino MJ, Stanick VA, Thostenson J, Kosten TR, Oliveto AH. Moderators of response to sertraline versus placebo among recently abstinent, cocaine dependent patients: A retrospective analysis of two clinical trials. Am J Addict. 2017; https://doi.org/10.1111/ajad.12635
	Chartier KG, Sanchez K, Killeen TK, Burrow A, Carmody T, Greer TL, et al. Men and Women From the STRIDE Clinical Trial: An Assessment of Stimulant Abstinence Symptom Severity at Residential Treatment Entry. Am J Addict. 2015; https://doi.org/10.1111/ajad.12190
	Chermack ST, Bonar EE, Ilgen MA, Walton MA, Cunningham RM, Booth BM, et al. Developing an Integrated Violence Prevention for Men and Women in Treatment for Substance Use Disorders. J Interpers Violence. 2017; https://doi.org/10.1177/0886260515586369
	Greenwood GL, Woods WJ, Guydish J, Bein E. Relapse outcomes in a randomized trial of residential and day drug abuse treatment. J Subst Abuse Treat. 2001; https://doi.org/10.1016/S0740-5472(00)00147-1
	Lowmaster SE, Morey LC, Baker KL, Hopwood CJ. Structure, reliability, and predictive validity of the Texas

	Christian University Correctional Residential Self-Rating Form at Intake in a residential substance abuse treatment facility. J Subst Abuse Treat. 2010; https://doi.org/10.1016/j.jsat.2010.05.002
	Marhe R, Luijten M, Van De Wetering BJM, Smits M, Franken IHA. Individual differences in anterior cingulate activation associated with attentional bias predict cocaine use after treatment. Neuropsychopharmacology. 2013; https://doi.org/10.1038/NPP.2013.7
	McLellan AT, Grissom GR, Zanis D, Randall M, Brill P, OBrien CP. Problem-service `matching' in addiction treatment - A prospective study in 4 programs. Arch Gen Psychiatry. 1997;54:730–5.
	Moos RH, King MJ, Burnett EG, Andrassy JM. Community residential program policies, services, and treatment orientations influence patients' participation in treatment. J Subst Abuse. 1997; https://doi.org/10.1016/S0899-3289(97)90015-8
	Silverman MJ. Effects of Music Therapy on Change Readiness and Craving in Patients on a Detoxification Unit. J Music Ther. 2011; https://doi.org/10.1093/jmt/48.4.509
	Trombello JM, Carmody T, Greer TL, Walker R, Rethorst CD, Trivedi MH. Psychosocial relationship status and quality as predictors of exercise intervention adherence and substance use outcomes: Results from the STRIDE (CTN-0037) study. PSYCHIATRY Res. 2017; https://doi.org/10.1016/j.psychres.2017.04.062
	Usdan SL, Schumacher JE, Milby JB, Wallace D, McNamara C, Michael M. Crack cocaine, alcohol, and other drug use patterns among homeless persons with other mental disorders. Am J Drug Alcohol Abuse. 2001; https://doi.org/10.1081/ADA-100103121
Studies not designed to predict treatment outcomes	Ball SA, Schottenfeld RS. A five-factor model of personality and addiction, psychiatric, and AIDS risk severity in pregnant and postpartum cocaine misusers. Subst Use Misuse. 1997; https://doi.org/10.3109/10826089709027298

	Batki SL, Canfield KM, Smyth E, Ploutz-Snyder R. Health-related quality of life in methadone maintenance patients with untreated hepatitis C virus infection. Drug Alcohol Depend. 2009; https://doi.org/10.1016/j.drugalcdep.2008.12.012
	Brooks KM, Castillo-Mancilla JR, Morrow M, MaWhinney S, Rowan SE, Wyles D, et al. Adherence to Direct-Acting Antiviral Therapy in People Actively Using Drugs and Alcohol: The INCLUD Study. OPEN FORUM Infect Dis. 2021; https://doi.org/10.1093/ofid/ofaa564
	Cornelius JR, Salloum IM, Thase ME, Haskett RF, Daley DC, Jones-Barlock A, et al. Fluoxetine versus placebo in depressed alcoholic cocaine abusers. Psychopharmacol Bull. 1998;34:117–21.
	Crits-Christoph P, Gallop R, Sadicario JS, Markell HM, Calsyn DA, Tang W, et al. Predictors and moderators of outcomes of HIV/STD sex risk reduction interventions in substance abuse treatment programs: a pooled analysis of two randomized controlled trials. Subst Abus Treat Prev POLICY. 2014; https://doi.org/10.1186/1747-597X-9-3
	Decker SE, Frankforter T, Babuscio T, Nich C, Ball SA, Carroll KM. Assessment Concordance and Predictive Validity of Self-Report and Biological Assay of Cocaine Use in Treatment Trials. Am J Addict. 2014; https://doi.org/10.1111/j.1521-0391.2014.12132.x
	del Palacio-Gonzalez A, Hesse M, Thylstrup B, Pedersen MU, Pedersen MM. Effects of contingency management and use of reminders for drug use treatment on readmission and criminality among young people: A linkage study of a randomized trial. J Subst Abuse Treat. 2022; https://doi.org/10.1016/j.jsat.2021.108617
	Dinis MML, Passos SRL, Camacho LAB. Predictive validity of the Brazilian version of the Expected Treatment Outcome Scale in cocaine-dependent outpatients at a drug treatment referral center. Rev Bras Psiquiatr. 2005; https://doi.org/10.1590/S1516-44462005000300012
	Doyle SR, Donovan DM. Applying an ensemble classification tree approach to the prediction of completion of

	a 12-step facilitation intervention with stimulant abusers. Psychol Addict Behav. 2014; https://doi.org/10.1037/A0037235
	Gelberg L, Robertson MJ, Arangua L, Leake BD, Sumner G, Moe A, et al. Prevalence, Distribution, and Correlates of Hepatitis C Virus Infection Among Homeless Adults in Los Angeles. Public Health Rep. 2012; https://doi.org/10.1177/003335491212700409
	Iovine AP, Drachman D, Kirane H. Risk Factors for Treatment Drop-Out: Implications for Adverse Outcomes When Treating Opioid Use Disorder. J Soc Work Pract Addict. 2020; https://doi.org/10.1080/1533256X.2020.1838859
	Kosten TR, Scanley BE, Tucker KA, Oliveto A, Prince C, Sinha R, et al. Cue-induced brain activity changes and relapse in cocaine-dependent patients. Neuropsychopharmacology. 2006; https://doi.org/10.1038/sj.npp.1300851
	Li Q, Chen X, Li X, Gorowska M, Li Z, Li Y. The Effects of Immediate vs Gradual Reduction in Nicotine Content of Cigarettes on Smoking Behavior: An Ecological Momentary Assessment Study. Front Psychiatry. 2022; https://doi.org/10.3389/fpsyt.2022.884605
	Maume MO, Lanier C, DeVall K. The Effect of Treatment Completion on Recidivism Among TASC Program Clients. Int J Offender Ther Comp Criminol. 2018; https://doi.org/10.1177/0306624X18780421
	Mills KL, Barrett EL, Merz S, Rosenfeld J, Ewer PL, Sannibale C, et al. Integrated Exposure-Based Therapy for Co-Occurring Post Traumatic Stress Disorder (PTSD) and Substance Dependence: Predictors of Change in PTSD Symptom Severity. J Clin Med. 2016; https://doi.org/10.3390/jcm5110101
	Morie KP, DeVito EE, Potenza MN, Worhunsky PD. Longitudinal changes in network engagement during cognitive control in cocaine use disorder. Drug Alcohol Depend. 2021; https://doi.org/10.1016/j.drugalcdep.2021.109151

	Murray RL, Chermack ST, Walton MA, Winters J, Booth BM, Blow FC. Psychological aggression, physical aggression, and injury in nonpartner relationships among men and women in treatment for substance-use disorders. J Stud Alcohol Drugs. 2008;69:896–905.
	Notzon DP, Mariani JJ, Pavlicova M, Glass A, Mahony AL, Brooks DJ, et al. Mixed-amphetamine salts increase abstinence from marijuana in patients with co-occurring attention-deficit/hyperactivity disorder and cocaine dependence. Am J Addict. 2016; https://doi.org/10.1111/AJAD.12467
	Nuijten M, Blanken P, van den Brink W, Hendriks V. Treatment of crack-cocaine dependence with topiramate: A randomized controlled feasibility trial in The Netherlands. Drug Alcohol Depend. 2014; https://doi.org/10.1016/j.drugalcdep.2014.02.024
	Nyamathi A, Stein JA, Schumann A, Tyler D. Latent variable assessment of outcomes in a nurse-managed intervention to increase latent tuberculosis treatment completion in homeless adults. Heal Psychol. 2007; https://doi.org/10.1037/0278-6133.26.1.68
	Nyamathi A, Salem BE, Zhang S, Farabee D, Hall B, Khalilifard F, et al. Nursing CaseManagement, Peer Coaching, and Hepatitis A and B Vaccine Completion Among Homeless Men Recently Released on Parole Randomized Clinical Trial. Nurs Res. 2015; https://doi.org/10.1097/NNR.0000000000000083
	Ramirez N, Arranz B, Salavert J, Alvarez E, Corripio I, Maria Duenas R, et al. Predictors of schizophrenia in patients with a first episode of psychosis. PSYCHIATRY Res. 2010; https://doi.org/10.1016/j.psychres.2009.03.013
	Rizkallah E, Mongeau-Pérusse V, Lamanuzzi L, Castenada-Ouellet SA, Stip E, Juteau LC, et al. Cannabidiol effects on cognition in individuals with cocaine use disorder: Exploratory results from a randomized controlled trial. Pharmacol Biochem Behav. 2022; https://doi.org/10.1016/j.pbb.2022.173376
	Schmitz JM, Oswald LM, Jacks SD, Rustin T, Rhoades HM, Grabowski J. Relapse prevention treatment for

	cocaine dependence: Group vs. individual format. Addict Behav. 1997; https://doi.org/10.1016/S0306-4603(96)00047-0
	Sexton MB, Dawson S, Spencer RJ, Phillips D, Reckow JM, Conroy DA, et al. Relationships between insomnia and alcohol and cocaine use frequency with aggression among veterans engaged in substance use treatment. Sleep Med. 2021; https://doi.org/10.1016/j.sleep.2020.10.010
	Siqueland L, Chittams J, Frank A, Thase ME, Gastfriend DR, Mercer D, et al. The protocol deviation patient: Characterization and implications for clinical trials research. Psychother Res. 1998;8:287–306.
	Xu J, Kober H, Wang X, DeVito EE, Carroll KM, Potenza MN. Hippocampal volume mediates the relationship between measures of pre-treatment cocaine use and within-treatment cocaine abstinence. Drug Alcohol Depend. 2014; https://doi.org/10.1016/j.drugalcdep.2014.07.007
Animal studies	Buffalari DM, Baldwin CK, See RE. Treatment of cocaine withdrawal anxiety with guanfacine: relationships to cocaine intake and reinstatement of cocaine seeking in rats. Psychopharmacology (Berl). 2012; https://doi.org/10.1007/s00213-012-2705-1
	Kromrey SA, Czoty PW, Nader SH, Register TC, Nader MA. Preclinical Laboratory Assessments of Predictors of Social Rank in Female Cynomolgus Monkeys. Am J Primatol. 2016; https://doi.org/10.1002/ajp.22514
	Paule MG. Using identical behavioral tasks in children, monkeys, and rats to study the effects of drugs. Curr Ther Res Exp. 2001; https://doi.org/10.1016/S0011-393X(01)80088-6
	Renda CR, Madden GJ. Impulsive choice and pre-exposure to delays: III. Four-month test-retest outcomes in male wistar rats. Behav Processes. 2016; https://doi.org/10.1016/j.beproc.2016.03.014
	Back SE, Gray K, Santa Ana E, Jones JL, Jarnecke AM, Joseph JE, et al. N-acetylcysteine for the treatment of

Studies reporting rationale and methods	comorbid alcohol use disorder and posttraumatic stress disorder: Design and methodology of a randomized clinical trial. Contemp Clin Trials. 2020; https://doi.org/10.1016/j.cct.2020.105961
	Crits-christoph P, Siqueland L, Blaine J, Frank A, Luborsky L, Onken LS, et al. The National Institute on Drug Abuse Collaborative Cocaine Treatment Study. Arch Gen Psychiatry. 1997; https://doi.org/10.1001/archpsyc.1997.01830200053007
	Dey M, Wenger A, Baumgartner C, Herrmann U, Augsburger M, Haug S, et al. Comparing a mindfulness- and CBT-based guided self-help Internet- and mobile-based intervention against a waiting list control condition as treatment for adults with frequent cannabis use: a randomized controlled trial of CANreduce 3.0. BMC Psychiatry. 2022; https://doi.org/10.1186/s12888-022-03802-9
	Forster SE, Forman SD, Gancz NN, Siegle GJ, Dickey MW, Steinhauer SR. Electrophysiological predictors and indicators of contingency management treatment response: Rationale and design for the ways of rewarding abstinence project (WRAP). Contemp Clin Trials Commun. 2021; https://doi.org/10.1016/j.conctc.2021.100796
	Mestre-Pintó JI, Fonseca F, Schaub MP, Baumgartner C, Alias-Ferri M, Torrens M. CANreduce-SP—adding psychological support to web-based adherence-focused guided self-help for cannabis users: study protocol for a three-arm randomized control trial. Trials. 2022; https://doi.org/10.1186/s13063-022-06399-2
	Moska C, Goudriaan AE, Blanken P, van de Mheen D, Spijkerman R, Schellekens A, et al. Youth in transition: Study protocol of a prospective cohort study into the long-term course of addiction, mental health problems and social functioning in youth entering addiction treatment. BMC Psychiatry. 2021; https://doi.org/10.1186/s12888-021-03520-8
	Schaub M, Sullivan R, Stark L. Snow Control - An RCT protocol for a web-based self-help therapy to reduce cocaine consumption in problematic cocaine users. BMC Psychiatry. 2011; https://doi.org/10.1186/1471-

	244X-11-153
	Schmitz JM, Stotts AL, Vujanovic AA, Weaver MF, Yoon JH, Vincent J, et al. A sequential multiple assignment randomized trial for cocaine cessation and relapse prevention: Tailoring treatment to the individual. Contemp Clin Trials. 2018; https://doi.org/10.1016/j.cct.2017.12.015
